# Parental investment matters for maternal and offspring immune defense in the mouthbrooding cichlid *Astatotilapia burtoni*

**DOI:** 10.1186/s12862-017-1109-6

**Published:** 2017-12-20

**Authors:** Isabel S. Keller, Walter Salzburger, Olivia Roth

**Affiliations:** 1 Evolutionary Ecology of Marine Fishes, GEOMAR - Helmholtz Centre for Ocean Research, Kiel, Germany; 2 Zoological Institute, University of Basel, Basel, Switzerland

**Keywords:** Parental care, Sexual dimorphism, Trans-generational immune priming, Immune system, Teleosts, Phenotypic plasticity, Gene expression

## Abstract

**Background:**

Parental care, while increasing parental fitness through offspring survival, also bears cost to the care-giving parent. Consequentially, trade offs between parental care and other vitally important traits, such as the immune system seem evident. In co-occurring phases of parental care and immunological challenges negative consequences through a resource allocation trade off on both the parental and the offspring conditions can be predicted. While the immune system reflects parental stress conditions, parental immunological investments also boost offspring survival via the transfer of immunological substances (trans-generational immune priming).

We investigated this relationship in the mouthbrooding East African cichlid *Astotatilapia burtoni*. Prior to mating, females were exposed to an immunological activation, while others remained immunologically naïve. Correspondingly, the immunological status of females was either examined directly after reproduction or after mouthbrooding had ceased. Offspring from both groups were exposed to immunological challenges to assess the extent of trans-generational immune priming. As proxy for immune status, cellular immunological activity and gene expression were determined.

**Results:**

Both reproducing and mouthbrooding females allocate their resources towards reproduction. While upon reproduction the innate immune system was impeded, mouthbrooding females showed an attenuation of inflammatory components. Juveniles from immune challenged mouthbrooding females showed downregulation of immune and life history candidate genes, implying a limitation of trans-generational plasticity when parents experience stress during the costly reproductive phase.

**Conclusion:**

Our results provide evidence that both parental investment via mouthbrooding and the rise of the immunological activity upon an immune challenge are costly traits. If applied simultaneously, not only mothers seem to be impacted in their performance, but also offspring are impeded in their ability to react upon a potentially virulent pathogen exposure.

**Electronic supplementary material:**

The online version of this article (10.1186/s12862-017-1109-6) contains supplementary material, which is available to authorized users.

## Background

Males and females differ in their strategies of how to transfer genetic material to the next generation during reproduction [82]. Males produce mobile sperm just big enough to carry the genetic material, while females produce comparably large eggs that contain all necessities for embryogenesis [84]. Important consequences of this anisogamy are a higher maternal investment per reproductive unit and sex-specific evolutionary best reproductive strategies [49, 93]. Because male fitness is limited by the number of mating events, males tend to primarily invest into the display of sexual signals such as ornaments to enhance their attractiveness [36]. Female fitness, on the other hand, is limited by the number of reproductive units [7]. According to Bateman’s principle, females are thus selected to prolong their life span [7, 20, 93], which can be achieved by a more efficient immune defence and pathogen evasion strategy [48, 65, 71, 75, 80]. This, in turn, leads to a sexual immune dimorphism, since the males’ investment into secondary sexual signals is often at the expense of investing into immune defense (in the form of a resource allocation trade-off) [10, 72, 78, 85].

Sex-specific evolutionary strategies also exist for the extent of parental care [49]. This can additionally challenge or, alternatively, compensate the imbalance in investment per reproductive unit between females and males. An increased parental investment reduces the prospect of remating and therefore impedes the overall reproductive success of males and females alike [34, 93]. Importantly, the caregiving sex is more limited in the number of reproductive units during its lifetime [93]. This implies that sexual immune dimorphism and parental investment, both fitness related traits, are intermingled. As consequence, parents face a resource allocation trade-off between investment in future reproduction and investment in self-maintenance and immune defense [47, 71]. Due to high energy expenditure during parental care, most vertebrates show immunosuppression, loss of energy stores, micronutrient depletion, glucocorticoid stress response and/or oxidative stress [13, 29, 66].

Parents may also transfer non-genetic information about their environmental experience to their offspring, which provides the opportunity for adaptive trans-generational phenotypic plasticity [46, 60]. Such parental effects can influence offspring development, and induce epigenetic changes triggering differential gene expression in the offspring [4, 41, 98]. As a cross-generational inducible defense strategy, parents transfer information about the concurrent pathogen assembly in the environment (trans-generational immune priming (TGIP)) [1, 33]. In vertebrates, TGIP enables coping with pathogens when the offspring immune system is not yet fully functional [32, 38]. This can induce a faster maturation and thereby enhance fitness [95]. Mechanisms how parents prime their offsprings’ immune system are manifold [88]. Transfer of immune components, such as immunoglobulin M (IgM), complement components, proteins and enzymes via the egg has already been shown in fishes [3, 11, 59, 87, 89].

In addition to a direct immunological transfer via the egg, immunological information can also be transferred from parent to offspring through intimate contact with immune reactive tissues, such as mucus [31, 67, 79]. In cichlid fishes such as the discus fish or tilapia, offspring are micro nipping mucus from the parental epidermis during the entire free-swimming stage [18, 44]. Transfer of immunity to the next generation may explain the induced immunological activity (IgM and antimicrobial peptides) in the parental mucus. In line with this, immune relevant components are passed on via mouthbrooding in tilapia [79] when eggs and fry are guarded in the buccal cavity of the parents in close contact with the parental mucosa [44]. Mouthbrooding is a rather costly parental investment trait, as it challenges the parental cardiac and the ventilation system resulting in higher osmoregulation [68] followed by a drop of parental body condition [35]. Mouthbrooding fishes are thus hypothesized to face a resource allocation trade-off between brooding and other life-history traits, among them the immune system. The close contact between parents and offspring and limited options for food-uptake during mouthbrooding makes cichlids an excellent system to study the costs of parental care for the parents’ immune system and the possibility for TGIP via the eggs and via the buccal mucosa.

In this study, we investigated the trade-off between parental care and immunological activation in the East African cichlid *Astatotilapia burtoni*, a maternal mouthbrooder inhabiting Lake Tanganyika and its surroundings [26, 90, 91]. *A. burtoni* is a model species for various questions in the field of evolutionary biology and development (brain and eye development [56]; hormonal, behavioral and phenotypic adaptation [23, 24, 39, 40, 42, 45, 92]; immune gene expression analysis upon challenge with *Vibrio anguillarum* [22]; as well as genomics and transcriptomics [5, 14, 76, 77].

The first part of this study was designed to assess the costs associated with mouthbrooding and reproduction and its effect on the capability of mounting an immune response in adults. Therefore, we assigned immune challenged and immunologically naïve female *A. burtoni* to either mouthbrooding, only reproduction without mouthbrooding, or neither reproduction nor brooding (‘no reproduction’). Immune challenged females are hypothesized to suffer a severe resource allocation trade-off between mounting an immune response and investing in reproduction and brooding. We thus expected a gradually decreasing immune response from ‘no reproduction’ over ‘reproduction only’ to ‘mouthbrooding’. To evaluate how maternal investment affects sexual immune dimorphism also naïve male immune status was examined. Males were hypothesized to having a lower immune competence than non-brooding females, however, with rising costs of parental investment (i.e. reproduction and mouthbrooding), whereas female immunological activity was expected to decrease, diminishing the difference between the sexes.

In the second part of the study, we focused on the offspring from immunologically challenged and naïve mothers. To this end, offspring were either artificially raised or mouthbred and then examined for their immunological activity to address the existence and specificity of TGIP via the buccal mucosa during mouthbrooding. We hypothesized that immune components are transferred from the mother to the offspring not only directly via the egg but also additionally during mouthbrooding. Juveniles raised in the absence of the female were supposed to be less immune competent than juveniles bred within the buccal cavity of the female. To assess if parents can transfer specific immune components about the concurrent pathogens in the environment, juveniles were vaccinated with either the same (homologous), a distinct (heterologous), or no bacteria isolate as their mothers were already immunologically exposed to. If TGIP is specific, offspring from challenged females should show a higher immune competence after challenge with the homologous bacteria than heterologous challenged offspring and offspring from naïve females. Mouthbrooding provides the opportunity for a prolonged transfer of immune components through the buccal mucosa during the whole larval development. Therefore, mouthbred offspring were supposed to show a higher immunological activity than artificially raised offspring.

## Methods

### I. Cost of mouthbrooding on *Astatotilapia burtoni* females immune defence & II. Cost of reproduction and influence on sexual immune dimorphism in *Astatotilapia burtoni*

**Fig. 1:**
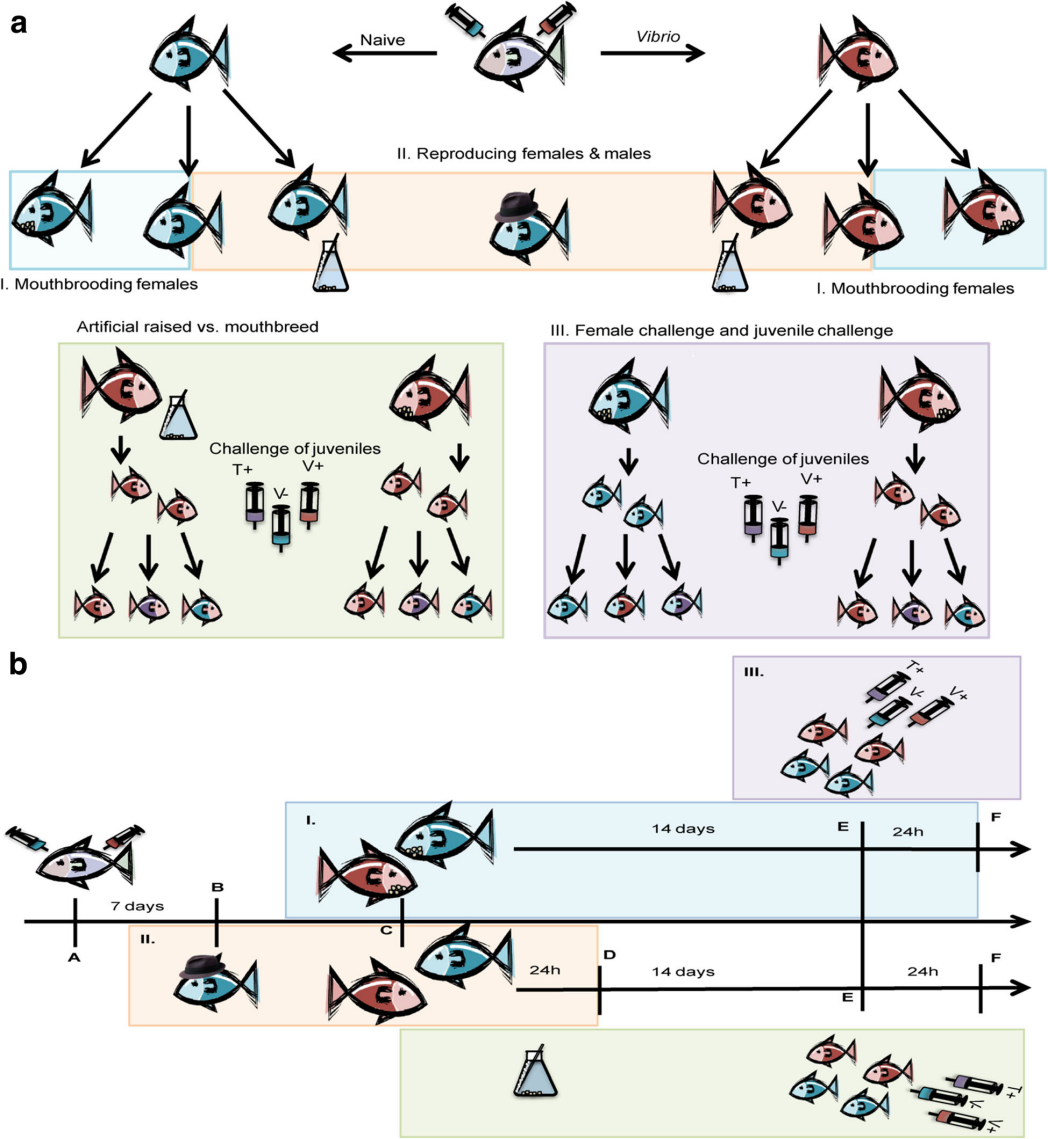
Experimental setup and timeline for adult and juvenile *A.burtoni*: **a** Experimental Setup. Challenged females (−) in red, naïve females (+) in blue, males wear a hat. Mouthbrooding females (B) carry eggs, reproducing-only females (R) are accompanied by a flask. Control females (C) are placed in between mouthbrooding and reproducing females. Smaller icons depict juveniles, color codes juvenile challenge (red: *V.anguillarum*, violet: *T.maritinum*, blue: PBS). Underlying color fit the respective statistics part: blue for cost of mouthbrooding on females (I.), red for cost of reproduction and sexual immune dimorphism (II.), violet for impact of female and juvenile challenge on mouthbred offspring (III.), green for comparison of artificially raised to mouthbred juveniles. **b** Timeline of the experiment. Color code, icons and roman lettering as above, controls are excluded. Letters show different time points of the experiment. A: Challenge of females, B: Introduction of males, C: Detection of brooding, split in either I. or II. D: Dissection of females from II. E: Release and challenge of juveniles, F: Dissection of females from I. and all juveniles

This experiment was designed to assess the costs of reproduction and mouthbrooding on the immune competence of females, and the impact of reproduction and mouthbrooding on a potential sexual immune dimorphism in the cichlid fish *Astatotilapia burtoni*. We immunologically challenged 35 female *Astatotilapia burtoni* by peritoneal injection of 50 μl of either 10^8^ heat-killed (65 °C for 60 min) *Vibrio anguillarum* (strain S6 M4, isolated from pipefish gut; (JQ598664 recombinase A (recA) gene partial cds) [73] diluted in PBS) (+, *n* = 18) or PBS (−, *n* = 17) as control. As the injected bacteria were heat-killed, this treatment served as an immunological activation similar to a vaccination. Hence, no clinical symptoms were observed and no animal died after the challenge. According to their immune challenge, females were tagged subcutaneously with Visible Implant Elastomer Tags (VIE; Northwest Marine Technology, Inc.; red and green fluorescent tags). After challenge and tagging, females were randomly placed in groups of three to four animals independent of their challenge in 80 l aquaria (from here on named “mating tanks”). Fish were held in in a circulation system at 26 °C with a 12 h day/12 h night light regime and fed daily with thawed out brine shrimp nauplii. One male per tank (1:3–4, male to female ratio) was introduced after allowing the females to acclimatize for one week. In the reproduction treatment (R), females were allowed to reproduce, but eggs were stripped the day after fertilization (6 females with priming (R+), 5 females naïve (R-)). In the control treatment (C), females were prevented from reproduction (7 females with priming (C+), 7 naïve females (C-)). In the brooding treatment (B), females were allowed to breed naturally until juveniles left the mouth of the female (7 females with priming (B+); 7 naïve females (B-)). At the first sign of egg uptake after fertilization, females were transferred to 10 l aquaria with one fish per tank in a climate chamber (set to 28 °C air temperature and resulting in 26 °C water temperature) and randomly assigned to one of three treatments: Females of the reproduction treatment (R) were immediately stripped off their eggs, challenged and sampled 24 h after challenge. Females of the brooding treatment (B) were kept in the 10 l aquaria until the free-swimming juveniles were released from the buccal cavity after about 14 days, challenged and sampled 24 h later. Four females (2× B- / 2× B+) lost their eggs during brooding and were excluded from the experiment, thus lowering sample size to 31 females (´reproduction´: 6 females with priming (R+), 5 females naïve (R-)), ´control´ (C): 7 females with priming (C+), 7 naïve females (C-), ´brooding´ (B) 5 females with priming (B+); 5 naïve females (B-)). Females of the control treatment were randomly chosen and transferred to the climate chamber and either sampled 24 h after transfer (control for the reproduction females) or 14 days after transfer (control for the brooding females). Males were sampled after the last female of the tank had been transferred to the climate chamber (Fig. 1 a and b, roman letters (I., II. & III.) guide through results and discussion).

### III. Impact of maternal immune challenge on mouthbreed *Astatotilapia burtoni*

Here, we examined the effects of mouthbrooding on the immune system of the offspring and tested for the existence and specificity of trans-generational immune priming (TGIP) in *A. burtoni* via the eggs and via the buccal mucosa during mouthbrooding. To examine TGIP, we vaccinated virgin females with either Phosphate buffered saline (PBS) (−) or heat-killed *Vibrio anguillarum* in PBS (+). The latter induces the maternal immune system simulating a potential infection. Juveniles from the brooding treatment (B) were naturally bred until they left the buccal cavity of the female after about 14 days. Clutches from females of the reproduction treatment were raised separately in a breeding apparatus designed to keep the eggs in motion and aerated through an indirect water flow until the yolk sack was used up completely (after about 14 days). All breeding chambers were located in the same 10 l aquaria. Both, artificially raised and naturally bred juveniles were grown for two weeks post fertilization and were then randomly divided in three groups for immune challenge by pricking them with a syringe. To test for possible effects of the maternal challenge (+/−) or the maternal treatment (R/B) on the juvenile immune gene expression, juveniles from each batch were split into three groups. Group one was pricked, homologously to the maternal challenge, with drops of 10^10^ heat-killed (65 °C for 60 min) *Vibrio anguillarum* (strain S6 M4 diluted in PBS); group two was challenged heterologous to the maternal challenge with drops of 10^10^ heat-killed (65 °C for 60 min) *Tenacibaculum maritimum* (diluted in PBS), and group three with 1 μl of PBS (Fig. 1 a and b, roman letters guide through results and discussion). As juveniles were too small to be tagged, they were held in 2 l aquaria according to their treatment and sampled the next day.

#### Fish handling

All fish were killed by bathing in an overdose of MS222 (according to animal welfare permit MELUR V 312–7224.121-19 (67–5/13), “komparative Vergleichsstudie von Immunantwort-Transfer von Eltern zu Nachkommen in Fischarten mit extremer Brutpflege”). In adult fish, we measured total length (TL), standard length (SL), and weight (W) of all adult fish in order to calculate a condition factor as a proxy for fitness (K=W/TL^3^). For the adult gene expression analyses gills were dissected and stored in RNAlater. The three main immunological organs of fish [96] were used for the assessment of the cellular immune measurements: blood was taken as a proxy for systemic infections and transportation way of pathogens; the head kidney as main lymphocyte proliferation organ; and the spleen as blood filtration and pathogen neutralization organ. Juvenile fish were measured for total length and weight. For the gene expression analysis, the head was separated from the rest of the body and stored in RNAlater. We could not measure cellular immune parameters in juveniles, as they were too small for organ dissection.

#### Cellular immune parameter analysis

In order to compare immune dynamics and activation of immune response among the different female treatments and challenges, Flow Cytometric measurements of cell population and adaptive immune cell proliferation were conducted. Measurements were done with a BD Accuri C6 Flow Cytometer® following the protocols described in Roth et al. [72] with modifications for cichlids described in Diepeveen et al. [22]. After dissection, spleen and head kidney were individually smashed through 40 μm cell sieves (Falcon) and suspended in 500 μl RPMI-1640 cell medium (Sigma-Aldrich, diluted with 5% distilled H_2_O). Blood was collected from the caudal vein and diluted in 500 μl RPMI-1640 cell medium. For cell population measurement, 75 μl of live cells in suspension were mixed with 50 μl Propidium Iodide (20 μg/ml, Roth) and measurements were taken immediately after. Cell size (Forward scatter, FSC) and cell complexity (Side scatter, SSC) of up to 10′000 life cell counts per sample were recorded on slow flow rate. Lymphocytes (smaller cells with low complexity) and monocytes (larger cells with higher complexity) were distinguished based on their scatter pictures on the basis of their distinct morphology. For cell cycle analysis, 75 μl of living cells in suspension were killed with 75 μl of 70% EtOH and stained with 50 μl Propidium Iodide. The cell mixture was measured for up to 20′000 individual cell counts on medium flow rate. Cells in a dividing stage of the cell cycle (S- or G_2_/M-phase) have approximately double the DNA content than cells in a resting stage of the cell cycle (G_1_-phase), allowing the discrimination of active and resting cells according to the measured emission of red fluorescence of the Propidium Iodide binding to the cellular DNA of each cell. Flow cytometric measurements were analysed using predefined gating in the BD Accuri C6 Software (Version 1.0.264.21).

#### Gene expression assays

RNA from juveniles and adult gill samples were extracted with RNeasy 96 Universal Tissue Kit (Qiagen) following the manufacturers protocol for vacuum extraction. RNA yield was measured by spectrometry (NanoDrop ND-1000; peQLab) and 300 ng/μl was used for reverse transcription with QuantiTect®Reverse-Transcription Kit (Qiagen). Some samples (adults: 2 females B+, 1 female R+, 2 females B-, juveniles: 2 juveniles V+ from V+ females, 2 juveniles V+ from naïve females, 2 T+ juveniles from naïve females, 8 naïve juveniles from naïve females) were excluded from the gene expression analysis due to low RNA yields. In order to design cichlid specific primers for immune genes, we blasted immune relevant teleost gene sequences against an *Astatotilapia burtoni* reference transcriptome [5]. Sequences of those genes were then uploaded in the web based Primer3 software (Version 4.0.0) for primer picking. Primers were tested for specificity and efficiency with RT qPCR using 5× HOT FIREPol® EvaGreen® qPCR Mix Plus (ROX) (Solis BioDyne). 48 specific primer pairs with efficiencies above 90% and standard curves with slopes of log quality vs. threshold cycle (Ct) between −3.5 and 3.2 were then selected for further analyses (list of all primers see Additional file 1: Table S1).

The gene-expression patterns of 48 immune-related genes were measured using a Fluidigm-BioMarkTM system based on 96.96 dynamic arrays (GE-Chip). For pre-amplification of target cDNA a mix of 2.5 μl TaqMan PreAmp Master Mix (Applied Biosystems), 0.5 μl of 500 nM combined primer pairs (diluted with TE Buffer) and 0.75 μl HPLC H_2_O was used for 1.4 μl of cDNA. Mixture was pre-amplified (1 × 10 min; 95 °C; 16× (15 s; 95 °C, 4 min; 60 °C)) and diluted 1:10 with low EDTA-TE Buffer. For the chip run a sample mix with 3.5 μl 2× SSo FastEvaGreen Supermix with low Rox (BioRad) and 0.37 μl 20× DNA binding Dye sample loading reagent (Fluidigm) on 3.3 μl of pre-amplified 1:10 diluted cDNA and an assay mix with 3.5 μl 2× Assay loading reagent (Fluidigm) and 3.15 μ 1× low EDTA-TE Buffer on 0.7 μl of 50 μM Primer mix have been prepared. 5 μl of each mix were loaded on a GE-chip, and measured with the GE-fast 96.96 PCR protocol in the BioMarkTM system according to Fluidigm instructions. In each Chip run we included two technical replicates, a negative control (HPLC H_2_O) and a –RT control to test for residual gDNA.

#### Data management & statistics

All statistical analyses were done in R version 3.1.3. GUI 1.65 Snow Leopard built (6912). All data were checked for normality and variance homoscedasticity. Wherever needed flow cytometric data were log transformed and gene expression data were cos (+20) transformed to fulfill assumptions for parametric testing.

When analyzing the adult data, we revealed differences among control animals kept in the climate chamber for short term (24 h; controls of the reproduction treatment) and those kept in the climate chamber for longer term (~14 days; controls of the brooding treatment). We thus had to split the adult data (flow cytometric measurements and gene expression of the gills) according to the location where the brooding (B)/ non-brooding (C) (I.) and reproduction (R)/ no reproduction (C) (II.) animals were kept. The controls for the brooding treatment and the mouthbrooding females (I.) were therefore analysed separately from the controls for the reproduction treatment and the reproduction only females and males (II.). All females used as control (C) were neither reproducing nor brooding. Our analysis is thus restricted to interpretations regarding ‘mouthbrooding’ versus ‘brooding control’ and ‘reproduction’ versus ‘reproduction control’, while mouthbrooding females cannot be directly compared to the reproduction only females. Males were sacrificed 24 h after their last reproductive event, handling was thus most similar to the reproduction females. Males are thus in the statistical comparison included in the comparison between reproduction and no reproduction (II.).

Due to high mortalities in juveniles reared artificially and descending from naïve reproduction only females (R-) (only 1 juvenile survived), we had to exclude all juveniles reared artificially from the analysis even though survival rate did not differ between the treatments (ANOVA of total juvenile number per female (naïve or challenged) at the end of two weeks mouthbrooding or artificial raising; F_3/17_ = 1.701, *p* = 0.205). Thus in the juvenile data set (III.), we only compared mouthbred juveniles from challenged females to those from naïve females, which permitted to determining the effect of maternal immune challenge on juvenile condition and to assessing the transfer of immunological information.

Cellular immune parameter data were composed to flow cytometric measurements of cell populations and cell proliferation of adult fish (I. & II.). Cell populations were measured as the relative proportion of lymphocyte (l) and monocyte (m) counts to the total of live cells. Cell proliferation shows the relative proportion of cells in dividing- (s) or in resting phase (r) to single cells in total. We calculated the proportion of both lymphocytes to monocytes (l/m) and dividing- to resting phase (s/r) for statistical analysis. Samples with a live cell count lower than 10% of total events were removed from the analysis. Data were analysed using an ANCOVA with the two factors treatment and challenge and the condition factor (K) as a covariate (aov(x~treatment*challenge + K)). For both adult datasets (I. & II.), the same model was used. Whether the random factor “mating tank” influences the results was tested in an initial ANOVA model. As the random factor was not significant, it was excluded from the final model. Tukey HSD (95% family-wise confidence levels) served as post hoc test if necessary.

Data from the gene expression analysis were processed using the Fluidigm-integrated software (Fluidigm Real-Time PCR analysis; BioMark Version 4.1.2). Samples with melt curves that deviated in mean temperature from the mean melt curve per gene were excluded. Mean cycle threshold (Ct), standard deviation (SD), and coefficient of variance (CV) were calculated for each remaining sample duplicate. Samples with a CV lower than 4% were replaced by the mean value over all samples per gene. One gene (*HA_PCAF; histone acetyltransferase*) was removed, as too many samples did not sufficiently match the criteria mentioned above. *HIVEP 3b* and *ADNPB* had the lowest geNorm (qbase + version 3.0, biogazelle) values, which indicates that they were most constant over all treatments, and were thus chosen as reference genes. For relative gene expression, the geometric mean of these two reference genes (*HIVEP 3b* and *ADNPB*) was subtracted from the mean Ct value of the gene of interest per sample resulting in ΔCt values. This was done for the gene expression data from juveniles and adults in the same way. Genes were grouped according to their function (GO terms; UniProt [6]) for multivariate statistics (Table 1).

**Table 1:** Division of candidate genes in functional groups

Gene Group			Gene Name	Gene Group	Gene Name
All Immune System genes	Adaptive Immune System		Fibronectin beta antigen CD29	Metabolism	Proprotein convertase subtilisin
			Fibronectin beta antigen CD81		Elongation Factor 1
			Ig light chain		Ribosomal protein A3
			Interleukin 10	Sex related genes	Androgen receptor A
			Integrin alpha 2		Androgen receptor B
			MHC I antigen F10 alpha chain		Aromatase B
			MHC II b	Epigenetic genes	DNA methyltransferase 1
			Lymphocyte cytosolic factor I		Histone acetyltransferase
			IgG FC binding protein		Histone deacetylase
	Innate immune system	Inflammation	Allograph inflammation factor		Histone demethylase
			Coagulation factor II /Thrombin		histone lysine methyltransferase
			Chemokine receptor 9		Lysine specific demethylase
			Lectine	Development	Myogenic regulatory factors
			Tumor necrose factor beta		Early growth response 1
		Oxidative Stress	Catalsase		Growth hormone rh
			Copper zink dismutase		Opsin 1
			Trypsin I	Stress	Heat shock protein 70
		Various innate IS	Calreticulin 3		Heat shock protein 90
			Calreticulin 1		*Glucocorticoid* receptor
			FAM60 A Protein		Heat shock protein 60
			Pentraxin 4	Reference	Activity-dep neuroprotector
			Serum amyloid A		Hivep 3b
		Complement Components	Complement component 1q		
			Complement component 9		
		Antimicrobial Peptides	Hepcidin		
			Latescidin 2		

Statistical analysis of adult gene expression was done calculating a PERMANCOVA with challenge and treatment as factors and condition factor (K) as covariable for each gene group (adonis(x~treatment*challenge + K, method = “euclidean”, permutations = 1000)). The same formula was applied for all adult datasets (brooding/non-brooding gills (I.) & reproduction/no reproduction gill (II.)). For significant PERMANCOVA factors, univariate analyses served as post hoc tests to identify the impact on each gene. These ANCOVAs used the same model (aov(x~treatment*challenge + K)) and a Tukey HSD test if necessary, to depict the direction of the differences among treatments (as done in [9]). To address the gene expression of juvenile cichlids (III.), we included family in the model, as some of the samples are siblings and therefore not independent we performed a nested MANOVA with female treatment nested in family [74]. Significant data were then post hoc tested in a nested ANOVA with the same factors as for the MANOVA (anova(x~jtreatment*ftreatment + ftreatment%in%family)). Further post hoc testing was done with Tukey HSD.

## Results

### I. Cost of mouthbrooding on *Astatotilapia burtoni* female immune defence

**Fig. 2:**
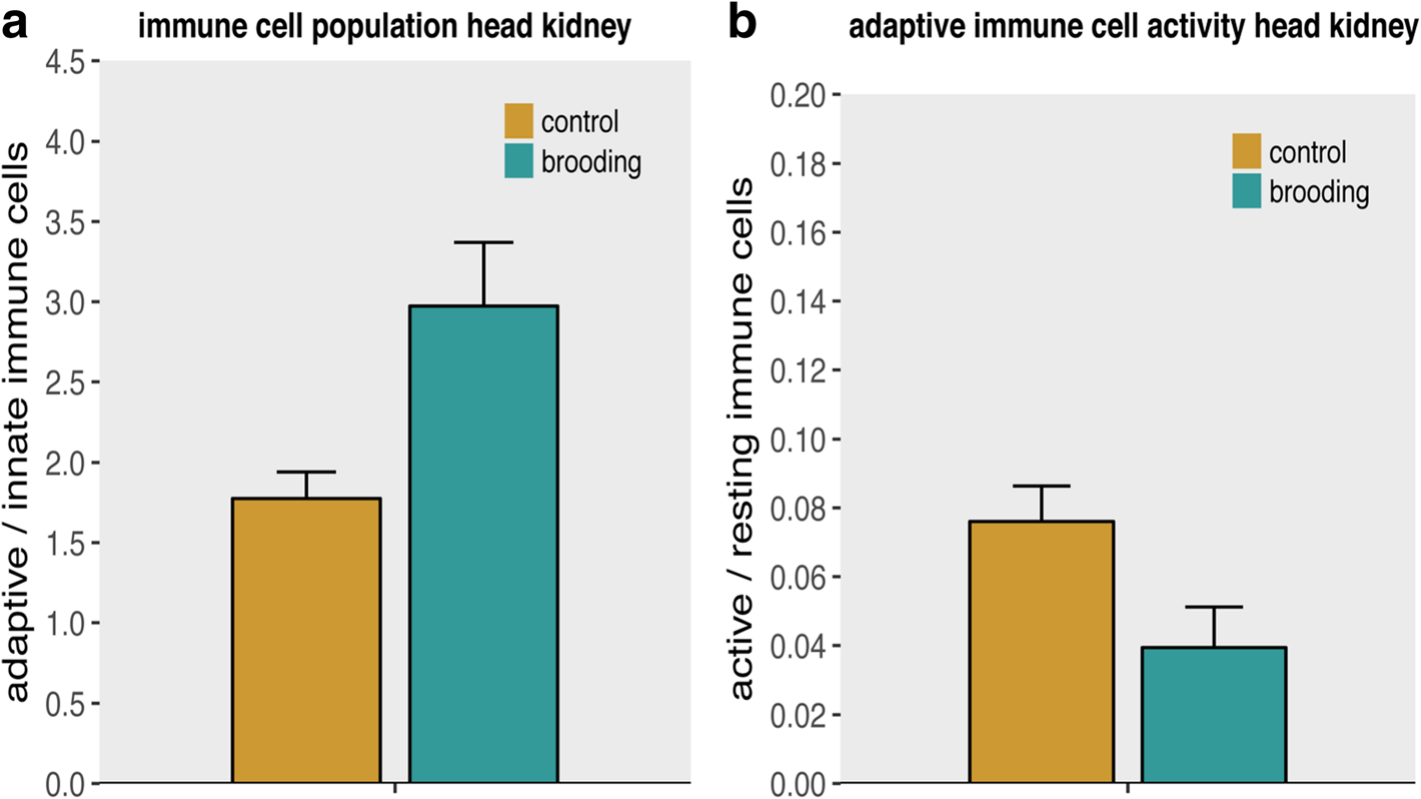
Immune cell measurements of brooding versus non-brooding females: Bars and error bars show group means with SE. Brown for control females; turquoise for mouthbrooding females. All shown differences are significant (*p* < 0.05). **a** Proportion of adaptive to innate immune cells of the head kidney. **b** Proportion of active to resting adaptive immune cells of the head kidney

To assess costs of mouthbrooding, challenged and naïve mouthbrooding females were compared to challenged and naïve control females, which were neither brooding nor reproducing. Brooding was successful in five of seven naïve and five of seven *Vibrio* challenged females (four females (2× B- /2× B+) lost their brood within two weeks). Mouthbrooding influenced both the proportion of adaptive to innate immune cells and the activity of the adaptive immune system in the head kidney. Brooding females had a higher proportion of adaptive to innate immune cells, whereas the proportion of active adaptive immune cells was lowered during brooding. Immune challenge had no effect on both cellular immune parameters (Table 2; Fig. 2 a and b).

**Table 2:** Two-way ANCOVA results of cellular immune parameter from brooding vs. non-brooding females: Significant *p* values (*p* < 0.05) are marked in bold letters. Results from Tukey HSD posthoc tests can be found in Additional file 2: Table S2

		Blood				Spleen				Head Kidney			
	Df	*SS*	*MS*	*F value*	*Pr(>F)*	*SS*	*MS*	*F value*	*Pr(>F)*	*SS*	*MS*	*F value*	*Pr(>F)*
Lymphocyte/Monocyte													
Treatment	1	0.017	0.017	0.89	0.378	0.70	0.70	2.11	0.190	4.30	4.30	5.62	**0.050**
Challenge	1	0.008	0.008	0.43	0.531	0.07	0.07	0.20	0.667	0.07	0.07	0.09	0.775
Condition factor	1	0.018	0.018	0.94	0.364	0.01	0.01	0.03	0.861	0.10	0.10	0.13	0.733
Treatment*Challenge	1	0.006	0.006	0.34	0.580	0.07	0.07	0.21	0.660	0.02	0.02	0.02	0.887
Residuals	7	0.132	0.019			2.32	0.33			5.37	0.77		
Active/Inactive Cells													
Treatment	1	0.000	0.000	3.32	0.1113	0.51	0.51	0.54	0.488	0.00	0.00	7.29	**0.031**
Challenge	1	0.000	0.000	0.00	0.9643	0.23	0.23	0.24	0.638	0.00	0.00	0.08	0.783
Condition factor	1	0.000	0.000	0.13	0.7311	1.10	1.10	1.17	0.316	0.00	0.00	5.84	0.046
Treatment*Challenge	1	0.000	0.000	0.01	0.9433	0.05	0.04	0.05	0.834	0.00	0.00	0.40	0.546
Residuals	7	0.000	0.000			6.63	0.95			0.00	0.00

**Fig. 3:**
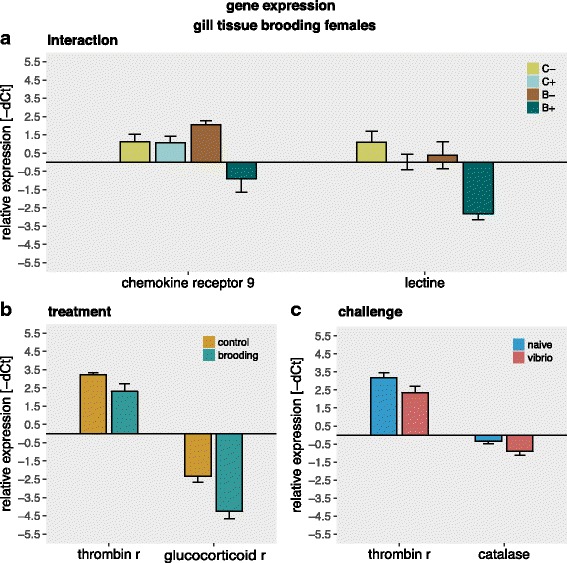
Gene expression of brooding versus non-brooding females: All graphs show relative expression of Ct values (-ΔCt), bars and error bars show group means with SE. Lettering denotes significance; only genes with effects (*p* > 0.05) are shown. Graphs are sorted according to the significant factor. **a** Interaction of treatment (brooding (B) and control (C)) and challenge (*Vibrio* (+) and naïve (−)) **b** Treatment effects of brooding (in turquoise) versus non-brooding (brown) **c** Challenge effects of *Vibrio* (red) and PBS (blue) challenge

Overall gene expression of the gill tissue was affected in three gene groups; inflammation genes (univariate effects in *lectine*, *chemokine receptor 9* & *thrombin receptor*), general innate immune system genes (univariate effects in *catalase*) and genes involved in stress response (univariate effects in *glucocorticoid receptor*). The interaction of mouthbrooding and immune challenge downregulated the expression of *lectine* and *chemokine receptor 9* compared to the naïve treatments (C- & B-) (Fig. 3a). Challenge alone (C+) had no effect on the expression of both *lectine* and *chemokine receptor 9*. Mouthbrooding females have a lower expression of both *thrombin receptor like 1* and *glucocorticoid receptor* (Fig. 3b). Immune challenge with *Vibrio* downregulated the expression of *thrombin receptor like 1* and *catalase* (Multivariate: Table 3; Fig. 3c; Univariate Additional file 2: Table S2, Tukey HSD: Additional file 3: Table S3).

**Table 3:** Two-way PERMANCOVA results of candidate gene expression from brooding vs. non-brooding females: PERMANCOVA to asses effects of treatment, challenge and their interaction on the relative expression of candidate genes (ΔCt values). A condition factor (K=W/TL^3^) was included as covariable. Significant results are marked in bold letters. Results of the univariate posthoc analyses (ANCOVA & Tukey HSD) can be found in Additional file 2: Table S2 and Additional file 3: Table S3

Gene categories	Model	Treatment (T)		Challenge (C)		K		T*C	
	*R* ^*2*^	*F Model*	*Pr (>F)*	*F Model*	*Pr (>F)*	*F Model*	*Pr (>F)*	*F Model*	*Pr (>F)*
All genes	0.62	0.93	0.507	0.93	0.499	1.28	0.261	1.11	0.326
All IS genes	0.62	0.74	0.608	0.94	0.453	1.45	0.208	1.08	0.349
Adaptive IS	0.67	0.57	0.619	0.48	0.708	1.68	0.216	0.76	0.489
All innate IS	0.50	1.46	0.186	2.42	**0.033**	1.50	0.188	1.71	0.117
Inflammation	0.19	6.92	**0.015**	12.28	**0.001**	4.09	**0.044**	5.69	**0.022**
Oxidative Stress	0.73	0.34	0.725	0.27	0.767	1.07	0.358	0.92	0.403
Various innate IS	0.59	0.22	0.796	1.97	0.178	1.25	0.303	1.33	0.280
Antimicrobial Peptides	0.52	2.59	0.101	1.04	0.421	1.76	0.207	1.21	0.374
Metabolism genes	0.70	0.88	0.431	0.87	0.463	0.52	0.666	0.69	0.555
Epigenetic genes	0.71	1.76	0.182	0.32	0.884	0.09	0.964	0.62	0.672
Developmental genes	0.66	0.68	0.519	0.45	0.616	0.82	0.376	1.60	0.235
Stress related genes	0.41	4.79	**0.005**	2.57	0.074	1.33	0.299	1.28	0.312
Complement Component	0.56	1.03	0.411	1.69	0.133	1.08	0.371	1.59	0.175
Sex related genes	0.71	1.76	0.182	0.32	0.884	0.09	0.964	0.62	0.672
Df Residuals / Model	7	1		1		1		1	
Df Total	11								

### II. Cost of reproduction and influence on sexual immune dimorphism in *Astatotilapia burtoni*

**Fig. 4:**
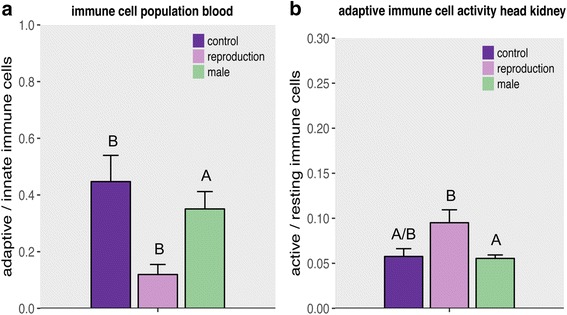
Immune cell measurements of males and reproducing versus non-reproducing females: Both graphs show bars and error bars with group means and SE. Lettering denotes significant differences. Females are shown in violet (dark for control, and light for reproduction) and males are shown in green. **a** Proportion of adaptive to innate immune cells of the blood. **b** Proportion of active to resting adaptive immune cells in the head kidney

Comparing immune challenged and naïve reproducing with immune challenged and naïve non-reproducing control females allows assessing the cost of reproduction and simultaneous immune challenge in *A. burtoni* females. By including naïve males in this comparison we are able to determine the influence of reproduction and female challenge on sexual immune dimorphism. The proportion of adaptive to innate immune cells in the blood of reproducing females was lower than in non-reproducing females and males. Females that had reproduced featured a lower proportion of resting cells in the head kidney than both non-reproducing females and males. The proportion of dividing cells, as well as the dividing to resting cell ratio in the head kidney did not differ between the two female treatments, but between reproducing females and males. A higher ratio of active to inactive adaptive immune cells indicates that reproduction induces the adaptive immune cell proliferation. *Vibrio* challenge had no effect on cellular immune parameters (Table 4; Fig. 4; Posthoc: Additional file 4: Table S4).

**Table 4:** Two-way ANCOVA results of cellular immune parameter from males, reproducing and non-reproducing females: Significant *p* values (*p* < 0.05) are marked in bold letters. Results from Tukey HSD posthoc tests can be found in Additional file 4: Table S4

		Blood				Spleen				Head Kidney			
	Df	*SS*	*MS*	*F value*	*Pr(>F)*	*SS*	*MS*	*F value*	*Pr(>F)*	*SS*	*MS*	*F value*	*Pr(>F)*
Lymphocyte/Monocyte													
Treatment	2	14.50	7.25	12.25	**<0.01**	1.85	0.92	2.71	0.093	1.73	0.87	1.79	0.194
Challenge	1	0.60	0.60	1.01	0.33	0.01	0.01	0.03	0.859	0.14	0.14	0.29	0.594
Condition factor	1	1.06	1.06	1.79	0.20	0.40	0.40	1.17	0.295	0.12	0.12	0.24	0.629
Treatment*Challenge	1	0.77	0.77	1.30	0.27	0.06	0.06	0.17	0.681	0.39	0.39	0.81	0.378
Residuals	19	11.24	0.59			6.12	0.34			9.17	0.48		
Active/Inactive Cells													
Treatment	2	2.08	1.04	2.11	0.15	13.05	6.53	1.82	0.188	1.48	0.74	4.65	**0.023**
Challenge	1	0.73	0.73	1.47	0.24	10.32	10.32	2.87	0.106	0.00	0.00	0.02	0.887
Condition factor	1	1.23	1.23	2.50	0.13	4.62	4.62	1.29	0.270	0.48	0.48	2.99	0.100
Treatment*Challenge	1	0.37	0.37	0.75	0.40	1.12	1.12	0.31	0.582	0.05	0.05	0.33	0.570
Residuals	19	8.38	0.49			71.78	3.59			3.02	0.16

**Fig. 5:**
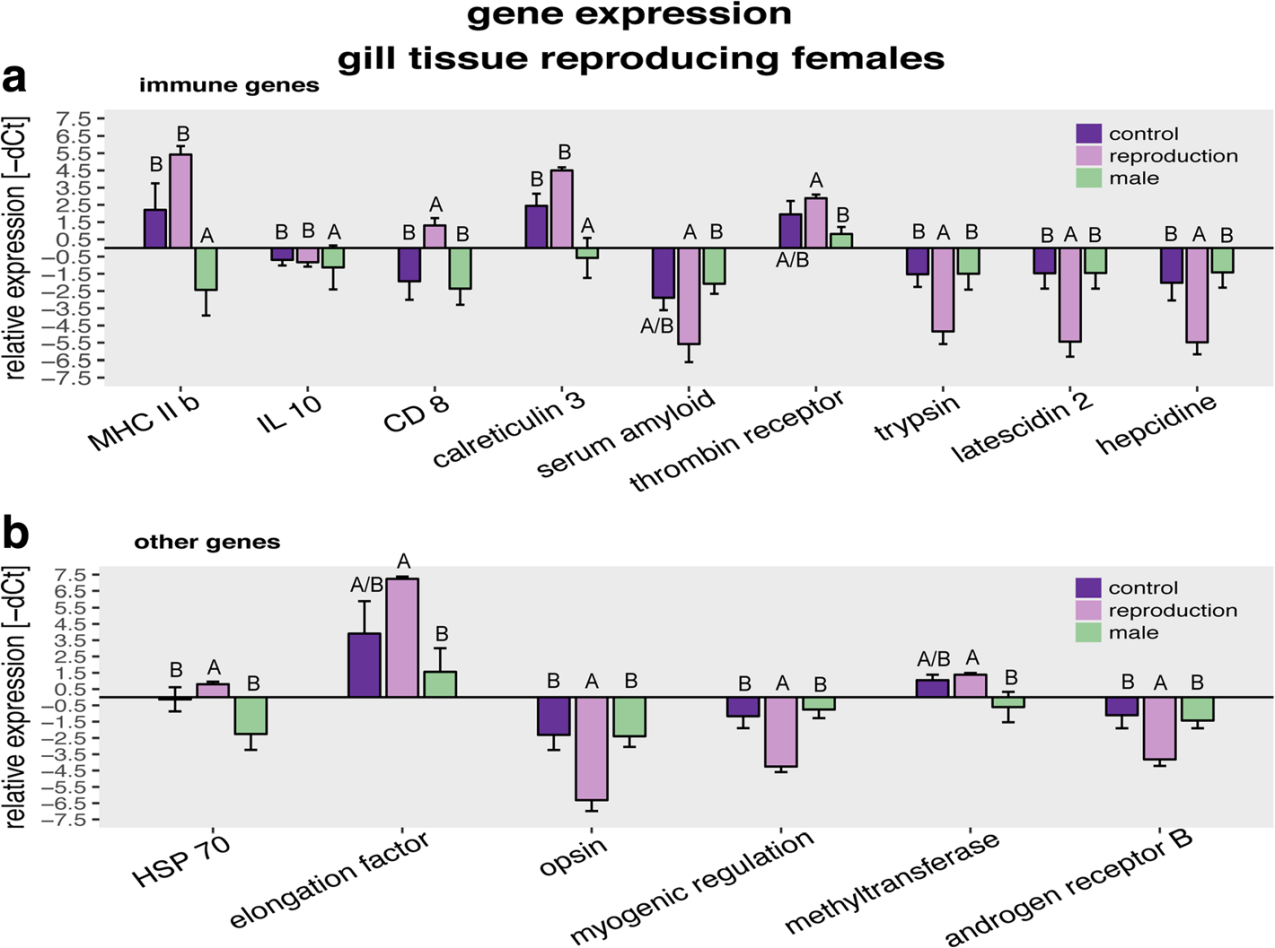
Gene expression of males and reproducing versus non-reproducing females: All graphs show relative expression of Ct values (-ΔCt), bars and error bars show group means with SE. Lettering denotes significance; only significantly different genes are shown. Females in violet (dark for control, and light for reproduction) and males in green. Graphs are sorted according to candidate gene function. **a** Genes of the immune system. **b** Genes from other gene groups

Multivariate analyses reflected that gene expression of the gill tissues was affected by reproduction but not by immune challenge in seven gene groups: “all genes”, “innate immune system genes & complement genes”, “adaptive immune system genes”, “antimicrobial genes & oxidative stress genes”, “developmental genes” and “metabolism genes”. In the following univariate analysis, 15 of 45 genes of interested showed differential expression between the treatments. In more detail, the univariate analysis revealed that the expression of *latescidin 2*, *hepcidin*, *trypsin 1*, *myogenic regulation factor*, *opsin 1* and *androgen receptor B* were downregulated in reproducing females compared to both other treatment groups. Expression of *CD81 antigen*, involved in cell proliferation and maturation of T- and B- cells, was upregulated during reproduction. Furthermore, reproducing females had a higher expression of *thrombin receptor like I*, *elongation factor 1* and *DNA methyltransferase* but a lower expression of the *serum amyloid A5 protein* gene, than males but not than control females. Males showed a lower expression of *MHC2b*, *heat shock protein 70*, *calreticulin 3* and *interleukin 10* than reproducing and non-reproducing females (Multivaraiate: Table 5; Fig. 5 A & B; Univariate: Additional file 5: Table S5; Tukey HSD: Additional file 6: Table S6).

**Table 5:** Two-way PERMANCOVA results of candidate gene expression from males, reproducing and non-reproducing females: PERMANCOVA to asses effects of treatment, challenge and their interaction on the relative expression of candidate genes (ΔCt values). A condition factor (K=W/TL^**3**^) was included as covariable. Significant results are marked in bold letters. Results of the univariate posthoc analysis (ANCOVA & Tukey HSD) can be found in Additional file 5: Table S5 and Additional file 6: Table S6

Permancova									
Gene categories	Model	Treatment (T)		Challenge (C)		K		T*C	
	*R2*	*F. Model*	*Pr (>F)*	*F. Model*	*Pr (>F)*	*F. Model*	*Pr (>F)*	*F. Model*	*Pr (>F)*
All genes	0.64	3.23	**0.002**	0.76	0.649	0.62	0.755	1.05	0.351
All IS genes	0.62	3.37	**0.003**	0.77	0.633	0.81	0.565	1.29	0.243
Adaptive IS	0.58	3.48	**0.001**	0.85	0.534	1.39	0.229	2.18	0.062
All innate IS	0.64	3.46	**0.003**	0.71	0.685	0.46	0.852	0.78	0.581
Inflammation	0.74	1.87	**0.105**	1.01	0.404	0.10	0.971	0.03	0.999
Oxidative Stress	0.66	2.92	**0.031**	0.77	0.480	0.34	0.736	0.88	0.423
Various innate IS	0.61	4.46	**0.003**	0.51	0.708	0.44	0.675	0.50	0.651
Antimicrobial Peptides	0.49	6.58	**0.011**	0.69	0.535	1.62	0.205	1.56	0.218
Metabolism genes	0.69	3.49	**0.049**	0.13	0.939	0.03	0.941	0.19	0.741
Epigenetic genes	0.70	0.93	0.493	2.16	0.063	0.22	0.918	0.88	0.464
Developmental genes	0.58	5.41	**0.006**	0.10	0.997	0.06	0.981	1.49	0.230
Stress related genes	0.75	1.19	0.331	0.98	0.398	0.89	0.457	0.36	0.811
Complement Component	0.65	3.31	**0.001**	0.72	0.663	0.48	0.840	0.79	0.559
Sex related genes	0.55	4.88	**0.003**	1.55	0.223	0.19	0.885	0.98	0.384
Df Residuals / Model	17	2		2		1		1	
Df Total	23

### III. Impact of immune challenge on mouthbred offspring

To determine the existence and specificity of trans generational immune priming via buccal mucosa in *A. burtoni* mouthbreed juveniles from either immune challenged (*Vibrio*) or naïve (PBS) females where challenged with the same (*Vibrio*, homologous challenge) or a different (*Tenacibacter,* heterologous challenge) heat-killed bacteria than the maternal challenge or left naïve in a fully reciprocal design. Both female and juvenile challenge impacted, in mouthbred offspring, the expression of genes involved in the innate immune system & complement component system, the adaptive immune system, but also epigenetic, sex related and developmental genes (Multivariate: Table 6).

**Table 6:** nested MANOVA results of gene expression analysis of mouthbred juveniles: nested MANOVA to asses effects of maternal challenge, juvenile challenge and their interaction on the relative expression of candidate genes (ΔCt values) in mouthbred juveniles. Female challenge was nested in family. Significant results are marked in bold letters. Results of the univariate posthoc analysis (ANCOVA & Tukey HSD) can be found in Additional file 7: Table S7 and Additional file 8: Table S8

nested MANOVA		All genes					All immune genes					Adaptive genes				
Factors	DF	Pillai	F	n Df	d Df	Pr(>F)	Pillai	F	n Df	d Df	Pr(>F)	Pillai	F	n Df	d Df	Pr(>F)
Juvenile Challenge (jC)	2	1.98	5.35	90	4	**0.055**	1.16	1.11	52	42	0.362	0.37	0.97	18	76	0.497
Maternal Challenge (mC)	1	1.00	2972.31	45	1	**0.015**	0.86	4.67	26	20	**< 0.001**	0.28	1.60	9	37	0.152
jC * mC	2	1.92	1.03	90	4	0.571	1.20	1.21	52	42	0.263	0.31	0.77	18	76	0.726
mC in family	4	3.95	6.87	180	16	**0.000**	2.85	2.20	104	92	**< 0.001**	1.24	2.01	36	160	**0.002**
Residuals	45															
		All innate IS					Inflammation					Oxidative Stress				
Factors	DF	Pillai	F	n Df	d Df	Pr(>F)	Pillai	F	n Df	d Df	Pr(>F)	Pillai	F	n Df	d Df	Pr(>F)
Juvenile Challenge (jC)	2	0.75	1.27	30	64	0.211	0.34	1.72	10	84	0.089	0.19	1.50	6	88	0.188
Maternal Challenge (mC)	1	0.80	8.45	15	31	**< 0.001**	0.28	3.25	5	41	**0.015**	0.05	0.68	3	43	0.568
jC * mC	2	0.58	0.88	30	64	0.643	0.25	1.18	10	84	0.318	0.13	1.05	6	88	0.398
mC in family	4	1.90	2.06	60	136	**< 0.001**	0.50	1.26	20	176	0.212	0.39	1.66	12	135	0.082
Residuals	45															
		Various innate IS					Antimicrobial Peptides					Complement Components				
Factors	DF	Pillai	F	n Df	d Df	Pr(>F)	Pillai	F	n Df	d Df	Pr(>F)	Pillai	F	n Df	d Df	Pr(>F)
Juvenile Challenge (jC)	2	0.29	1.84	8	86	0.080	0.22	2.84	4	90	**0.029**	0.77012	1.105	34	60	0.361
Maternal Challenge (mC)	1	0.15	1.88	4	42	0.132	0.06	1.30	2	44	0.283	0.80387	6.992	17	29	**< 0.001**
jC * mC	2	0.14	0.84	8	86	0.571	0.05	0.59	4	90	0.667	0.64951	0.8487	34	60	0.693
mC in family	4	0.61	2.04	16	180	**0.013**	0.25	1.58	8	90	0.143	2.19249	2.2833	68	128	**< 0.001**
Residuals	45															
		Epigenetic genes					Developmental genes					Stress related genes				
Factors	DF	Pillai	F	n Df	d Df	Pr(>F)	Pillai	F	n Df	d Df	Pr(>F)	Pillai	F	n Df	d Df	Pr(>F)
Juvenile Challenge (jC)	2	0.25	1.19	10	84	0.306	0.23	1.40	8	86	0.208	0.28	1.76	8	86	0.097
Maternal Challenge (mC)	1	0.31	3.73	5	41	**0.007**	0.16	1.93	4	42	0.122	0.01	0.13	4	42	0.972
jC * mC	2	0.37	1.90	10	84	0.056	0.21	1.27	8	86	0.268	0.19	1.10	8	86	0.374
mC in family	4	0.70	1.88	20	176	**0.017**	0.54	1.74	16	180	**0.042**	0.29	0.89	16	180	0.586
Residuals	45															
		Metabolism genes					Sex related genes									
Factors	DF	Pillai	F	n Df	d Df	Pr(>F)	Pillai	F	n Df	d Df	Pr(>F)					
Juvenile Challenge (jC)	2	0.13	1.02	6	88	0.418	0.29179	2.5053	6	88	**0.027622**					
Maternal Challenge (mC)	1	0.17	2.91	3	43	**0.045**	0.2595	5.0231	3	43	**0.004501**					
jC * mC	2	0.06	0.49	6	88	0.816	0.12935	1.0142	6	88	0.421418					
mC in family	4	0.32	1.35	12	135	0.196	0.20872	0.8412	12	135	0.608014					
Residuals	45															

**Fig. 6:**
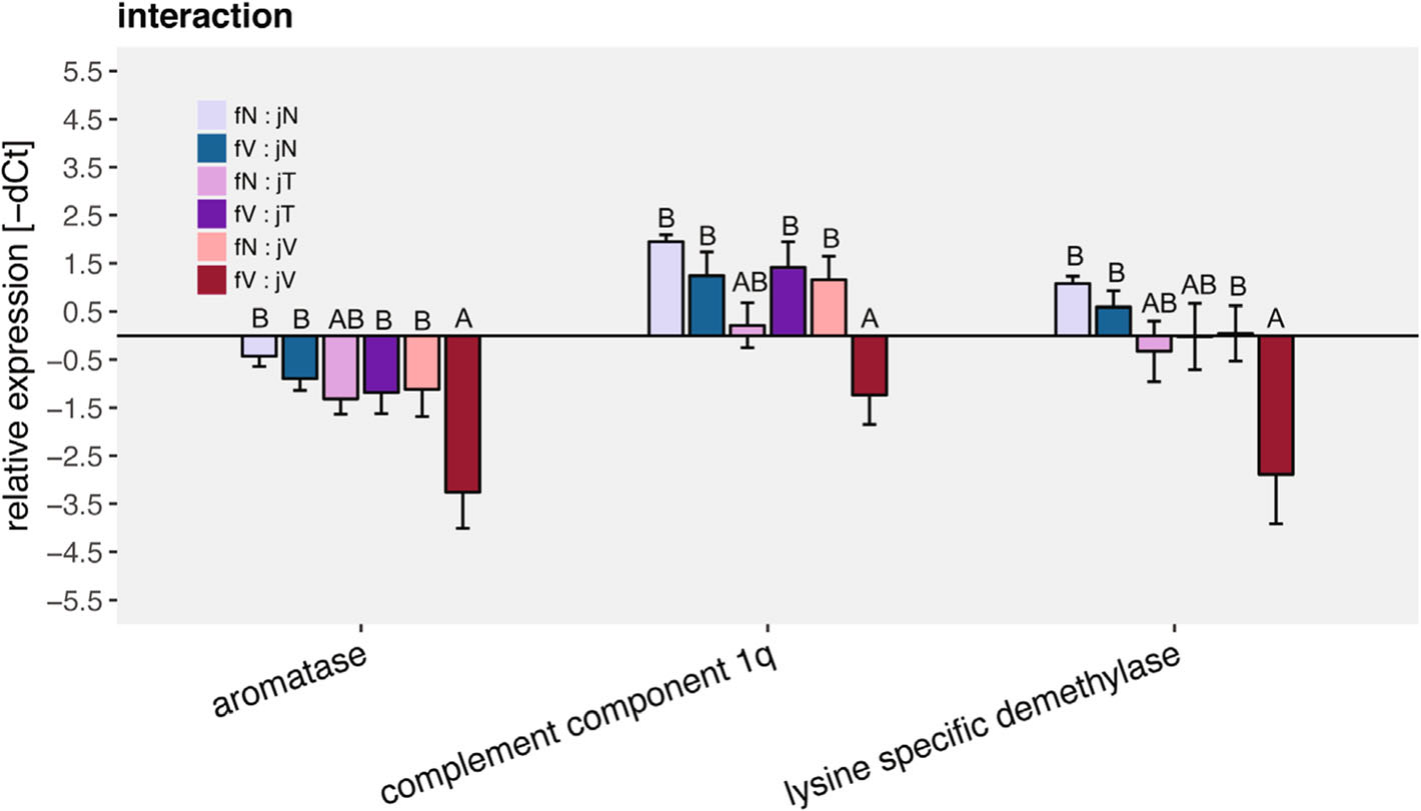
Gene expression of mouthbred juveniles; maternal challenge x juvenile challenge: All graphs show relative expression of Ct values (-ΔCt), bars and error bars show group means with SE. Lettering denotes significance; only significantly different genes are depicted. Abbreviation consist of female challenge and juvenile challenge (maternal challenge: juvenile challenge): fN for naïve maternal challenge, fV for maternal *Vibrio* challenge, jN for naïve juvenile challenge, jT for juvenile *Tenacibaculum* challenge, jN for juvenile *Vibrio* challenge

**Fig. 7:**
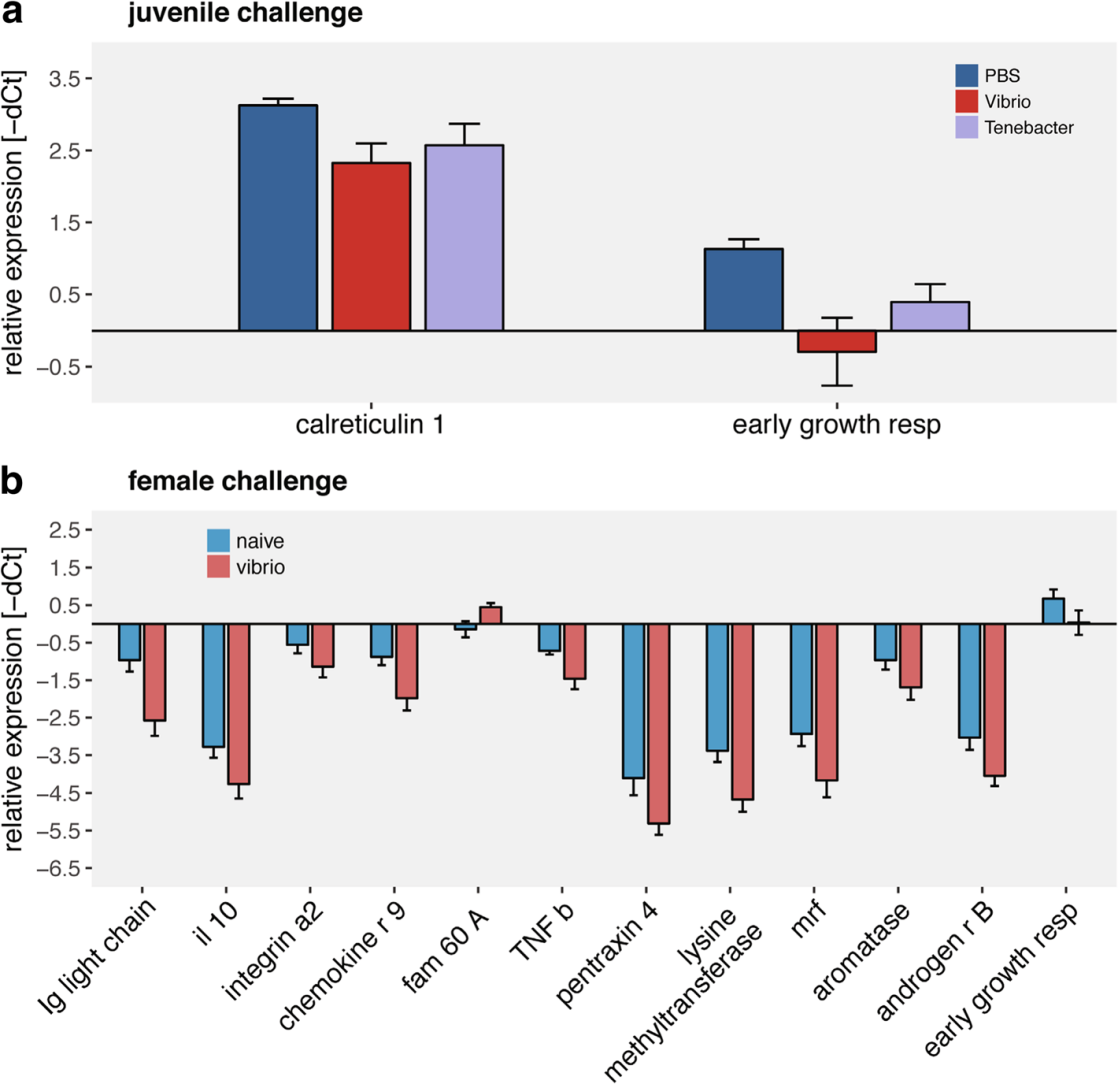
Gene expression of mouthbred juveniles; maternal challenge and juvenile challenge: All graphs show relative expression of Ct values (-ΔCt), bars and error bars depict group means with SE. Graphs are sorted according to significant factors. Lettering denotes significance; only genes with effects are shown. **a** Genes affected by juvenile challenge. Blue for naïve (PBS), red for *Vibrio* and violet for *Tenacibacter*. **b** Genes affected by maternal challenge. Light blue; naïve mothers (PBS) and light red; *Vibrio* challenged mothers

Single gene univariate analyses of those gene groups showed interactive effects of both female and juvenile immune challenge on *lysine specific demethylase* and *Aromatase B*. In both genes *Vibrio* challenged juveniles from *Vibrio* challenged females (fV:jV) had a lower expression than other juveniles (fN:jN, fN:jV, fV:jN) except those being challenged with *Tenacibaculum* (fN:jT & fV:jT). The expression of *complement component 1q* (*C1q*) was also lower in *Vibrio* challenged juveniles from *Vibrio* challenged females (fV:jV) as compared to all but *Tenacibaculum* challenged juveniles from naïve females (fN:jT) (Fig. 6). Juvenile challenge with *Vibrio* downregulated the expression of *calreticulin 1* (innate immune system) and early growth factor (developmental genes) compared to naïve juveniles (PBS) (Fig. 7a). Effects of female challenge on juvenile gene expression could be shown in 12 of 45 genes of interest. Juveniles of challenged females show a lower expression of *chemokine*, *interleukin 10, ig light chain*, *tumor necrosis factor b*, *integrin a2*, *pentraxin 4*, *myogenic regulation factors*, *early growth factor*, *histone lysine methyltransferase*, *aromatase B* and *androgen receptor B*. Only the expression protein *FAM 60A* was upregulated in juveniles from challenged females compared to juveniles from naïve females (Fig. 7b; Univariate: Additional file 7: Table S7; Tukey HSD: Additional file 8: Table S8).

## Discussion

While, to our knowledge, previous studies on TGIP have exclusively focused on either the parental costs or the offspring benefits [8, 18, 31, 67, 74, 79], this study aimed to illuminate the impact and the interaction of parental investment on the parental and the offspring immune system. The integration of both sides, parents and offspring, allows drawing conclusions about trade-offs in reproduction and immune defense. By enlightening consequences on both the parental and the offspring side, we can add to the understanding of costs and benefits of parental investment, immune response and the evolution of mouthbrooding in particular.

### I. Cost of mouthbrooding on *Astatotilapia burtoni* female immune defense

The first part of this study was designed to assess the costs associated with mouthbrooding and reproduction and their effect on the capability of mounting an immune response in adults. To this end, females of *A. burtoni* being challenged with heat-killed *Vibrio* bacteria or left naïve (challenge with PBS) were either allowed to complete mouth brooding (I.) or were stripped off their brood after reproduction (II.). Both treatment groups were then compared to non-reproducing individuals.

Mouthbrooding females showed a lowered activity of adaptive immune cells and their ratio of adaptive to innate immune cells was higher in the head kidney (Fig. 2); inflammation genes were downregulated, whereas stress related genes were upregulated (Fig. 3). Two genes, *chemokine receptor 9* (*CCR9*) and *lectin* were downregulated in females that had to pay the dual costs of mouthbrooding and activation of the immune system upon immune challenge. *CCR9* is involved in T-cell maturation and migration [94] and upregulated after *Vibrio anguillarum* infection in sea bass [30]. Lectin enhances the antibacterial and antifungal properties of mucus [54]. A downregulation of *CCR9* and *lectin* upon mouthbrooding in combination with immune challenge indicates a resource-allocation trade-off between parental care and the immune system. *Thrombin receptor* (*TR*) that is closely associated with the lectin activated complement pathway was downregulated during brooding and upon an immune challenge (Fig. 3). In previous studies with rock bream, higher *TR* expression has been found upon immune challenge with *Vibrio* [17].

Brooding and immune challenge may induce different stress responses. Moutbrooding led to a downregulation of *glucocorticoid receptor*. A correlation between reduction of *glucocorticoid receptors* and increased corticosterone secretion was identified in prenatally challenged rats [69]. Downregulation of *glucocorticoid receptor* could thus indicate higher cortisol levels. To our knowledge, cortisol levels during mouthbrooding has not yet been measured in cichlids. In *Oreochromis mosambicus*, a mouth brooding tilapine cichlid, treatment with cortisol decreased oozyte size and parental growth, indicating a trade-off between reproduction and somatic maintenance induced by cortisol [28]. Despite the fact that evidence of elevated stress in the parental phase of cichlids exists [43] concluding from reduced glucocorticoid receptor expression in the gills to higher general stress levels could be misleading. Nevertheless, glucocorticoid receptors are known to be involved in the anti-inflammatory response [16, 86]. Immune challenge led to a downregulation of *catalase*, an oxidative stress related gene. Low catalase levels may indicate lower antioxidant capacities, which could raise additional costs for the female, as brooding and reproduction were previously shown to elevate oxidative stress and lower the antioxidant capacities of the immune system in many organisms [2, 61].

In summary, our results suggest that parental care provided during mouthbrooding may be traded off with investment into the immune system. Mouthbrooding decreased the expression of *glucocorticoid receptor* thus possibly inducing the level of the stress hormone cortisol. Immune challenge elevated the extent of oxidative stress as reflected by a lower expression of catalase. Both, glucocorticoid receptor and catalase activity might additionally modulate the female immune system [37, 97].

### II. Cost of reproduction and influence on sexual immune dimorphism

We hypothesized a trade-off between investment in immune defense and reproduction in female mouthbrooding cichlid fish in the form of immune suppression during reproduction, as has previously been demonstrated in various vertebrate species [12, 21, 58, 63, 81]. To this end, we compared immune gene expression and cellular immune parameters of reproducing and non-reproducing females after either being challenged with *V. anguillarum* or left naïve. We indeed observed a reduced proportion of adaptive immune cells in the blood of reproducing females, irrespective of whether or not an immune challenge had taken place (Fig. 4), accompanied by the downregulation of genes of the innate immune system (*trypsin, latescidin 2, hepcidin*), some metabolism genes (*opsin 1, myogenic regulation factor*) and a hormone receptor (*androgen receptor B*) compared to non reproducing females (Fig. 5). However, we also found that the stress responsive *heat shock protein 70* (*HSP70*) and the *transmembrane protein CD81* (*CD81*) were upregulated in reproducing females. In the case of mouthbrooding cichlid fish, *HSP70* – on the basis of its protein chaperoning functions [19, 57, 70] – might be responsible for the transport of proteins into the buccal mucus to support brooding. In mouse it has been shown that HSP70 plays an important role in the maturation of dendritic cells and stimulates cytotoxic T-cell maturation via MHC class I [57]. Upregulation of *HSP 70* might possibly be triggered by a general physiological stress response, such as reproduction. *CD81* is a transmembrane protein, in complex with other responsible for both B- and T-cell maturation and proliferation (reviewed in Lewi et al. [50]). In mice it has been found to be expressed on MHC I molecules [15]. Due to the very diverse function of CD81, an upregulation can have several effects, depending on tissue and costimulatory molecules [50].

Regardless of their reproductive state, females showed a higher expression of adaptive immune genes, when compared to males (Fig. 5). This suggests that also haplochromine cichlid fishes exhibit a sexual immune dimorphism, with females having an elevated adaptive immunological baseline activity prolonging their lifespan to reach a similar reproductive output as males [71]. On the other hand, our data are in contrast to the resource allocation hypothesis, as we found that reproducing females have a higher adaptive immune cell activity (Fig. 4) and a higher expression of innate, developmental and epigenetic genes combined with a lower expression of acute phase protein (Serum Amyloid A5 (SAA)) than males but not than non-reproducing ‘control’ females. This contradicts previous results, where differences between reproductive and non-reproductive females were observed [12, 21, 25, 63]. One explanation for the induced adaptive immune response at reproduction could be that females invest into offspring immunity via TGIP, e.g. via the aggregation of proteins advantageous for the offspring in the newly formed egg [100]. The synthesis of such proteins could induce gene expression in the female. An interactive effect of reproduction and challenge on female gene expression, showing a clear pattern of TGIP or resource allocation trade-off, was not observed in the gill tissue.

Both, mouthbrooding and reproduction independently suppress parts of the female immune system and enhance stress responses in female *Astatotilapia burtoni*. Additionally, reproduction accentuates the present sexual immune dimorphism. Due to experimental constraints, we were not able to directly compare immune competence between reproducing and mouthbrooding females. Nevertheless, it seems that differential limitations are opposed on the female immune system during reproduction and mouthbrooding. Reproduction influenced mainly genes involved in metabolism and general innate immune system genes possibly due to extended energy expenditure during oogenesis. Mouthbrooding seems to induce stress reflected in a downregulation of inflammation responses and an increase of oxidative stress in the females. Additionally, mouthbrooding and reproduction differentially affect both the proportion and the activity of adaptive immune cells. These differences possibly arise due to differential allocation of resources in egg production and provisioning of larvae.

### III. Impact of immune challenge on mouthbred offspring

We aimed to address the transfer of immune components via the buccal mucosa as a potential additional immunological boost of offspring early life stages to the transfer via egg, and the specificity of such transferred immunological information. As opposed to our initial aim, we could not differentiate among transfer of maternal immunity via the eggs and via buccal mucosa, as mortality in the artificially bred offspring was too high. The much higher rate of survival in the treatment group where parental investment was provided implies that mouthbrooding is beneficial. We thus only discuss the differences in gene expression after homologous or heterologous immune challenge of offspring being mouthbred by *Vibrio* challenged or naïve females. Indications for such specific trans-generational immune priming would be identified via the interaction of female and juvenile challenge effects (Fig. 6). In contrast to our expectations, the *lysine specific demethylase* (*LSDM*), the *complement component 1q* (*C1q*) and *aromatase* (*Arom B*) were downregulated in juveniles treated with a challenge homologous to their mothers compared to the other treatments. *LSDM* is important for cell proliferation, embryonic development and transcription activity of T cells [52, 64]. *C1q* interacts with pattern recognition, inflammation and activation of the adaptive immune system [27] and is suggested to play a role in organ development [96]. *Aromatase* is responsible for the conversion of testosterone to estradiol and, if downregulated induces testosterone concentration in the organism, which potentially can be immune suppressive [55, 58]. This may indicate that females, which have the dual cost of inducing their immune system upon an immune challenge and providing parental investment via mouthbrooding, are limited in the resources they can invest into the eggs and larvae. This would be in line with the hypothesized resource-allocation trade-off between reproduction and the immune system and potentially imply that offspring from challenged females might be of lower quality than offspring of naïve females, reflected in their disability of upregulating gene expression upon an immune challenge. Alternatively, if females provide all necessary compounds via the egg or the buccal mucosa to the offspring, there may simply not be the need for offspring to upregulate those genes due to adaptive maternal effects. Offspring challenged with *Vibrio*, irrespective of the maternal challenge, had a lower expression of the *early growth receptor* (*egr1*), important in cell proliferation and embryogenesis of the gill vessel system [99]. *Vibrio* challenged offspring also had a lower expression of *calreticulin 1* (*calret 1*), responsible for protein chaperoning as response of oxidative stress [53] (Fig. 7). These results are in contrast to the current literature, as in this juvenile developmental stage and under infection both genes were shown to be upregulated [53, 99]. Downregulation of both genes could be a sign for lower developmental potential of the juveniles faced with a *Vibrio* challenge. *FAM60A* was upregulated in the offspring from the treatment group in which the mothers were immune challenged (Fig. 7). *FAM60A* regulates the expression of the TGF beta signaling pathway, increases cell migration and is, within a histone acetylation complex, responsible for elevated cell division during stress [62, 83]. Both the effects of the juvenile challenge and the maternal challenge on the offspring gene expression could be a sign of induced stress in the offspring from challenged mothers, previously shown to suffering elevated stress levels. Higher levels of stress hormones possibly suppress the offspring immune competence [51] and may even impede embryonic development. Due to the candidate gene approach taken, the probability is high that key genes responsible for both TGIP and specific defenses upon bacterial exposure were not captured in this study. With the limited number of genes assessed, we could not detect an adaptive pattern of trans-generational immune priming, however an impact of maternal immunological and stress experience. In contrast to our expectations, homologous maternal and offspring immunological exposure did not induce but rather downregulate the expression of genes involved in the complement system and in epigenetic regulation. This either implies that previous specific maternal immune challenge boosted the immunological response in the offspring such, that juveniles are not in need to induce the expression of several immune genes. Alternatively, maternal immune challenge impaired a specific activation of immune response, possibly due to a maternal resource allocation trade-off between reproduction and the maternal immune system. This implies that trans-generational phenotypic plasticity may be limited if concurrently to the reproductive event a stressor is met in the parental generation.

## Conclusion

Both the onset of reproduction and the long-lasting mouthbrooding are stressful for female cichlid fish due to the costs involved in the extreme parental investment provided. Shortly after fertilization females of the reproduction treatment reduce their innate immune response, metabolism, and hormone production, whilst genes involved in immune regulations and stress responses (e.g. *HSP70, CD81*) become upregulated. The possible preparation of the buccal mucosa for later brooding at this early stage of reproduction might be reflected in an induced activity of adaptive immune cells, and the enhanced expression of developmental and epigenetic genes, in particular in comparison to the lower immunological activity of males. However, when faced with an immune challenge, the investment into the buccal mucosa might be impeded due to high energy demands of the immune system, resulting in a resource allocation trade-off between reproduction and the immune system and potentially even a lower quality of offspring. At the end of mouthbrooding, when parental investment is ceased, females seem dissipated, which is reflected in their reduced expression of inflammation genes and an induced stress gene expression. The strong effect of maternal challenge on juveniles suggests the existence of maternal effects; nevertheless, no signs for adaptive trans-generational immune priming were detected. As a consequence, mothers exposed to an immune challenge that simultaneously fulfilled the task of brooding produced offspring with lower immune gene expression, implying a limited transfer of resources from stressed mothers towards their offspring. Parental investment boosts offspring survival. However, the energy requirements for parental investment are high and in a situation where other life-history traits may demand a reallocation of resources, the limits of energy availability seems reached. This may impede both mothers and offspring simultaneously, resulting in physiological stress on the maternal side and a reduced ability for activation of gene expression on the offspring side.

## Additional files

Additional file 1: Table S1. List of all primers used for candidate gene expression: Table depicts gene names, functions, fwd and rev sequences and references for those, which have not been designed by the authors. (PDF 43 kb)

Additional file 2: Table S2. ANCOVA on candidate genes from brooding versus non-brooding females: Univariate analysis following significant gene groups in multivariate PERMANCOVA. Significant *p*-Values are marked with asterisk (code: *p*-Value >0.001 ***; > 0.01 **; > 0.01*). *P*-values marked additionally in bold are in agreement with the results from the multivariate analysis. (PDF 57 kb)

Additional file 3: Table S3. Tukey HSD test on candidate genes from brooding versus non-brooding females: Posthoc test following significant results from two-way ANCOVA. *P*-values marked in bold are in agreement with the results from the univariate analysis. (PDF 32 kb)

Additional file 4: Table S4. Tukey HSD test on cellular immune parameter from males, reproducing and non-reproducing females: Posthoc test following significant results from two-way ANCOVA. *P*-values marked in bold are in agreement with the results from the univariate analysis. (PDF 32 kb)

Additional file 5: Table S5. ANCOVA on candidate genes from males, reproducing and non-reproducing females: Univariate analysis following significant gene groups in multivariate PERMANCOVA. Sigificant p-Values are marked with asterisk (code: *p*-Value >0.001 ***; > 0.01 **; > 0.01*). P-values marked additionally in bold are in agreement with the results from the multivariate analysis. (PDF 69 kb)

Additional file 6: Table S6. Tukey HSD test on candidate genes from males, reproducing and non-reproducing females: Posthoc test following significant results from two-way ANCOVA. *P*-values marked in bold are in agreement with the results from the univariate analysis. (PDF 41 kb)

Additional file 7: Table S7. nested ANOVA on candidate genes from mouthbred juveniles: Univariate analysis following significant gene groups in multivariate nested MANOVA. Significant p-Values are marked with asterisk (code: *p*-Value > 0.001 ***; > 0.01 **; > 0.01*). P-values marked additionally in bold are in agreement with the results from the multivariate analysis. (PDF 74 kb)

Additional file 8: Table S8. Tukey HSD test on candidate genes from mouthbred juveniles: Posthoc test following significant results from two-way ANCOVA. P-values marked additionally in bold are in agreement with the results from the univariate analysis. (PDF 51 kb)

## Abbreviations

B: Brooding treatment
C: Control treatment
FSC: Forward Scatter
K: condition factor
L: lymphocyte
M: Monocyte
MS222: Ethyl 3-aminobenzoate methanesulfonate
PBS: phosphate buffered saline
R: Reproduction treatment
R: Resting phase
S: Dividing phase
SL: standard length
SSC: Side Scatter
T: Tenebacilum treatment
TGIP: trans-generational immune priming
TL: total length
V: Vibrio treatment
VIE: Visible Elastomer Tags
W: weight
