# Supplementary material for: Parental investment matters for maternal and offspring immune defense in the mouthbrooding cichlid Astatotilapia burtoni
Source: BMC Evol Biol. 2017 Dec 20;17:264. doi: 10.1186/s12862-017-1109-6 (PMC5738712; doi:10.1186/s12862-017-1109-6)
Supplement: Supplementary file 1 — List of all primers used for candidate gene expression: Table depicts gene names, functions, fwd and rev sequences and references for those, which have not been designed by the authors. (PDF 43 kb) [file 12862_2017_1109_MOESM1_ESM.pdf]

S1 Table: List of all primers used for candidate gene expression:

Primer Name	Gene Name	Sequence		
		Forward	Reverse	
CD29	Fibronectin beta antigen CD29	CCTGCCTCCCTCAACAGTTATG	GTAGGACAGGATGGGGATGTTC	
CD8	Fibronectin beta antigen CD82	CAGTAAAGACAGCGCCATCAAG	GTAGGACATAAGGTGGTGGCAA	
igl	Immunoglobulin light chain	TTGGTGCCAATGTCAGAATCCT	TCTGGCTGATCAAATCCCACTG	
Il10	Interleukin 10	TGGAATTTTACCTGGACACGGT	CTGGGTGACATCTCTCTTCAGG	
integrin a2	Integrin alpha 2	TGGAAGCTAGGCATTTTACTGGA	CAAAGTCAGTGCAAATACTCCACA	
MHC1	MHC I antigen F10	GTGGTTGTTGGGATGGTTGATG	CTGATCTGTGTTCCTGGCAAAC	
MHC2b	Major histocompatibility complex II beta	AATCACCATCTGCCATCTCCTC	GCCCTATGTCAAACTTCACTCC	
LCP	Lymphocyte cytosolic factor I	TGATGGCAATGCTACCTTGACT	GTTCACCCAGCGTACAATGATG	
FcGr2A	Fc fragment of IgL	CCACAAAGGTGAAACTGACCC	AGGCTCTTGTGTACGTGTGG	
AIF	Allograph inflammation factor	GAGGTGGTTGGAAGTAGCTCAA	TGCCATGCCTTCAAACATCAAG	
F2RL1	coagulation factor II (Thrombin)	TGGCAATCTGGGTCTTTCTCTT	CATAGATCCAGTTGTTGCCGTT	
chemokine	Chemokine receptor 9	CCTTTGTGGTCTGCACGATGG	GATCTTCGGCACCTCCCTCT	
lectine	Lectine	TCTGCTTGGATCACAGTGGTAC	TCCACCTGCTGTAAAGTCTCAC	
TNFb	Tumor necrose factor beta	CCAGAGATGGCTTGTACAGTGT	TGTGGATCGATGAGAGGAGAGT	
catalase	Catalsase	CGCTGAACAAGAAAGACACCTG	CCATCCTTCATCCATTCCCAGA	
copzidis	Copper zink dismutase	TGTTGCCAAGATTGACATCACG	CGGTCTTCAGGCTCTCTTCATT	
FDF6	Trypsin I	GTCTCTGAACTCTGGCTACCAC	CTCAGTTCCCTCATTGACCGAA	
Calret 3	Calreticulin 3	TCTGGGAGTCTGGAGGATGATT	GTCCTCAGGCTTTGTGTCACTA	
Calret 1	Calreticulin 1	ATGAGATGGATGGAGAGTGGGA	GGTTGTCAATCTCAGGGTGGAT	
FAM 60A	FAM 60 A Protein	GCCAGATCAACAGGCTTCAG	CTGTGGGCATCTGAGTTGTG	
ptx4	Pentraxin 4	GAACCATGCAGAAGAGGTCAGA	CATGGAGTTGGTCTGGCTTAGT	
SAA	Serum amyloid A	CCCAATTGAAGCAGCTAAAGGG	TGTGCAGCATCAGAGTTTCCT	
C1q	Complement component 1q	CAAACATTCCCTGGTGTTCGAC	TGTTGAGGTTGTAGCTCGTCTC	
C9	Complement component 9	GACGAAGGTAAGGCTGAAGGAA	AAGGTCCATCACTCCATCCCTA	
hepcidin	Hepcidin	GGAGCCAATGAGCATGGACTAT	CTCAGAACCTGCAGCAAATTCC	
lat2	Latescidin 2	CGTCGTGCTCACATTCATTTGT	ACGCTTGTGTCTGCTCGTTATA	
PCSK2	Proprotein convertase subtilisin	GGAGAAGGGCTCTTTCACCT	GTTCGATGACATTCTTCACCCTT	Baldo et al. 2011
EF1	Elongation Factor 1	GCCCCTGCAGGACGTCTA	CGGCCGACGGTACAGT	Colombo et al. 2013
rspa3	Ribosomal protein A3	AGACCAATGACCTGAAGGAAGTG	TCTCGATGTCCTTGCCAACA	Colombo et al. 2013
ArA	Androgen receptor A	CGCTGTATCTGGTACGGTAG	TGAGGAATCGCACTTGG	Diepeveen et al. 2013

ArB	Androgen receptor B	TTCGGCGACAAGTACAACTC	ACTGTTCACGGCGCATTA	Diepeveen et al. 2013
AromB	Aromatase B	TGGAGGAGATGAACACCGTCT	TGTCGTTGTCCTCCAGAGCTT	Diepeveen et al. 2013
dmap1	DNA methyltransferase 1	AGTACCAAATGCATCTCCACGA	TTGCTGGTGATCATATCGGTCA	
HA_PCAF	histone acetyltransferase	GGTTGCATTTTGTGACCTCTCC	ACAAACGGAGGATCAGACATGG	
HD_AC1	Histone deacetylase	ATGGCGCTTACTTCTCAAGGAA	TCGCTTTGTGTGGACGGTATAT	
HDem_TRP	Histone demethylase	GTTCGCTTGGGTGACACAATTA	ACAAAGTGGTATCGTGCAGCTA	
EHMT2	Euchromatic histone lysine methyltransferase	ACTACAGTCGTCACCATGTCTG	CTGACAGGTCTTTCAGGCATCT	
LSDM	Lysine specific demethylase	GCGAATACAACGGCTCAATGAG	CAAAACCACCCTCTTCGTTCAC	
MRFs	Myogenic regulatory factors	CACAGCTTCCAATCCAAACCAG	GCTTCCTGAGTCACTCCCATAG	
egr1	Early growth response 1	TATACAGGCAGCTTCACCCTTG	AGGAGGATGAGATGGTCTGTGA	
ghrh	Growth hormone releasing hormone	TGCCAGAAAGTTCCTTCAGACA	ATTTCTCTGGATGGCTGTGAGG	
opn1sw	Opsin 1	CACTTCCACCTGTACGAGAACA	CGCCACGAGAACAATGAAGTTT	
HSP70	Heat shock protein 70	AGTTTCATGGCAGGGCATTTTC	TCCTCCTCCATGTACATCACCT	
HSP90	Heat shock protein 90	ACATTATGGAGGAGGAGGTGGA	AGGACTCAAGTTTGGTGGGATC	
CR	Cortisol receptor	ATGCTGGCAGAGATCATTTCCA	AAGGGACGGGATTTAAGGCATT	
HSP60	Heat shock protein 60	ACCATCATCAACGAGCTGAAGA	ATGCAGAGTCTTCCCATCCTTG	
ADNPB	activity-dependent neuroprotector homeobox	CGTCGAGAATGGTTTTGGACC	GCTTCTTGCTGATGATGGGAC	Baldo et al. 2011
HIVEP3b	Hivep 3b	CCAGACAGCCACAGCAACAA	CCCCACATGTTCCACATTCA	Diepeveen et al. 2013

Baldo L, Santos ME, Salzburger W. Comparative transcriptomics of eastern African cichlid fishes shows signs of positive selection and a large contribution of untranslated regions to genetic diversity. Genome Biol Evol. 2011;3(1):443–55.

Colombo M, Diepeveen ET, Muschick M, Santos ME, Indermaur A, Boileau N et al. The ecological and genetic basis of convergent thick-lipped phenotypes in cichlid fishes. Mol Ecol. 2013;22;670-684

Diepeveen ET, Roth O, Salzburger W. Immune-Related Functions of the Hivep Gene Family in East African Cichlid Fishes. G3 (Bethesda). 2013 Oct 18;
