# Supplementary material for: Parental investment matters for maternal and offspring immune defense in the mouthbrooding cichlid Astatotilapia burtoni
Source: BMC Evol Biol. 2017 Dec 20;17:264. doi: 10.1186/s12862-017-1109-6 (PMC5738712; doi:10.1186/s12862-017-1109-6)
Supplement: Supplementary file 2 — ANCOVA on candidate genes from brooding versus non-brooding females: Univariate analysis following significant gene groups in multivariate PERMANCOVA. Significant p-Values are marked with asterisk (code: p-Value >0.001 ***; > 0.01 **; > 0.01*). P-values marked additionally in bold are in agreement with the results from the multivariate analysis. (PDF 57 kb) [file 12862_2017_1109_MOESM2_ESM.pdf]

S2 Table: ANCOVA on candidate genes from brooding versus non-brooding females:
Univariate analysis following significant gene groups in multivariate PERMANCOVA. Sigificant p-Values are marked with asterisk (code: p-Value > 0.001 ***; >0.01 **; >0.01*). Bold **p-values** are in agreement with the multivariate analysis

ANCOVA aov(x~treatment*challenge+K)

**Allograph inflammation factor**	Df	SS	MS	F value	Pr(>F)	
Treatment	1	0	0	0	0.9928	
Challenge	1	0.0006	0.0006	0.002	0.9634	
Condition factor	1	2.1656	2.1656	7.69	**0.0276**	*
Treatment*Challenge	1	0.0328	0.0328	0.116	0.743	
Residuals	7	1.9714	0.2816			
**Chemokine receptor 9**	Df	SS	MS	F value	Pr(>F)	
Treatment	1	0.835	0.835	1.621	0.24357	
Challenge	1	6.768	6.768	13.141	**0.00845**	**
Condition factor	1	3.426	3.426	6.652	**0.03652**	*
**Treatment*Challenge**	1	4.554	4.554	8.842	**0.0207**	*
Residuals	7	3.605	0.515			
**Coagulation factor II / Thrombin**	Df	SS	MS	F value	Pr(>F)	
Treatment	1	2.5447	2.5447	11.139	**0.0125**	*
Challenge	1	2.0823	2.0823	9.115	**0.0194**	*
Condition factor	1	0.4833	0.4833	2.116	0.1891	
Treatment*Challenge	1	1.2581	1.2581	5.507	0.0513	.
Residuals	7	1.5991	0.2284			
**Lectine**	Df	SS	MS	F value	Pr(>F)	
Treatment	1	9.366	9.366	14.864	**0.00625**	**
Challenge	1	13.815	13.815	21.925	**0.00225**	**
Condition factor	1	1.279	1.279	2.03	0.19722	
Treatment*Challenge	1	4.626	4.626	7.342	**0.03022**	*
Residuals	7	4.411	0.63			
**Tumor necrose factor**	Df	SS	MS	F value	Pr(>F)	
Treatment	1	0.0318	0.03183	0.167	0.695	
Challenge	1	0.0042	0.00419	0.022	0.886	
Condition factor	1	0.203	0.20301	1.063	0.337	
Treatment*Challenge	1	0.0321	0.03208	0.168	0.694	
Residuals	7	1.3365	0.19092			
**Calreticulin 1**	Df	SS	MS	F value	Pr(>F)	
Treatment	1	0.7877	0.7877	4.513	0.07127	.
Challenge	1	0.2106	0.2106	1.206	0.30838	
Condition factor	1	2.8827	2.8827	16.513	0.00479	**
Treatment*Challenge	1	0.5215	0.5215	2.987	0.12754	
Residuals	7	1.222	0.1746			
**Calreticulin 3**	Df	SS	MS	F value	Pr(>F)	
Treatment	1	0.037	0.037	0.153	0.70722	
Challenge	1	0.31	0.31	1.274	0.29614	
Condition factor	1	3.275	3.275	13.469	0.00796	**
Treatment*Challenge	1	0.014	0.014	0.057	0.81819	
Residuals	7	1.702	0.243			
**Catalase**	Df	SS	MS	F value	Pr(>F)	
Treatment	1	0.7915	0.7915	5.346	0.054	.
Challenge	1	0.9415	0.9415	6.36	**0.0397**	*
Condition factor	1	0.0938	0.0938	0.633	0.4522	

Treatment*Challenge	1	0.1375	0.1375	0.929	0.3673	
Residuals	7	1.0363	0.148			
**FAM 60A Protein**	Df	SS	MS	F value	Pr(>F)	
Treatment	1	0.1151	0.1151	5.893	0.0456	*
Challenge	1	0.0881	0.0881	4.511	0.0713	.
Condition factor	1	0.4253	0.4253	21.783	0.0023	**
Treatment*Challenge	1	0.0068	0.0068	0.348	0.5739	
Residuals	7	0.1367	0.0195			
**Copper zink dismutase**	Df	SS	MS	F value	Pr(>F)	
Treatment	1	0.003	0.0027	0.005	0.944	
Challenge	1	0.101	0.1005	0.199	0.669	
Condition factor	1	1.62	1.6198	3.205	0.117	
Treatment*Challenge	1	1.64	1.6401	3.245	0.115	
Residuals	7	3.538	0.5055			
**Trypsin 1**	Df	SS	MS	F value	Pr(>F)	
Treatment	1	0.514	0.514	0.162	0.7	
Challenge	1	0.001	0.001	0	0.989	
Condition factor	1	2.395	2.395	0.753	0.414	
Treatment*Challenge	1	1.767	1.767	0.556	0.48	
Residuals	7	22.255	3.179			
**Hepcidin**	Df	SS	MS	F value	Pr(>F)	
Treatment	1	5.337	5.337	3.739	0.0944	.
Challenge	1	2.518	2.518	1.764	0.2258	
Condition factor	1	0.902	0.902	0.632	0.4528	
Treatment*Challenge	1	3.456	3.456	2.421	0.1637	
Residuals	7	9.991	1.427			
**Latescidin 2**	Df	SS	MS	F value	Pr(>F)	
Treatment	1	2.153	2.153	1.466	0.265	
Challenge	1	0.489	0.489	0.333	0.582	
Condition factor	1	4.19	4.19	2.852	0.135	
Treatment*Challenge	1	0.039	0.039	0.027	0.875	
Residuals	7	10.284	1.469			
**Pentraxin**	Df	SS	MS	F value	Pr(>F)	
Treatment	1	0.134	0.1337	0.075	0.792	
Challenge	1	0.774	0.7739	0.433	0.531	
Condition factor	1	0.044	0.0443	0.025	0.879	
Treatment*Challenge	1	2.273	2.2732	1.273	0.296	
Residuals	7	12.501	1.7859			
**Serum Amyliod A**	Df	SS	MS	F value	Pr(>F)	
Treatment	1	0.24	0.243	0.05	0.83	
Challenge	1	9.89	9.889	2.017	0.198	
Condition factor	1	0.11	0.112	0.023	0.884	
Treatment*Challenge	1	6.56	6.556	1.338	0.285	
Residuals	7	34.31	4.902			
stress related genes						
**Heat shock protein 60**	Df	SS	MS	F value	Pr(>F)	
Treatment	1	0.034	0.034	0.11	0.7499	
Challenge	1	3.1606	3.1606	10.232	0.0151	*
Condition factor	1	0.1842	0.1842	0.596	0.4652	
Treatment*Challenge	1	2.3365	2.3365	7.564	0.0285	*
Residuals	7	2.1622	0.3089			
**Heat shock protein 70**	Df	SS	MS	F value	Pr(>F)	
Treatment	1	0.6447	0.6447	3.566	0.1009	

Challenge	1	1.0332	1.0332	5.715	0.0481	*
Condition factor	1	1.349	1.349	7.462	0.0293	*
Treatment*Challenge	1	0.4402	0.4402	2.435	0.1626	
Residuals	7	1.2655	0.1808			
**Heat shock protein 90**	Df	SS	MS	F value	Pr(>F)	
Treatment	1	1.441	1.4411	0.952	0.362	
Challenge	1	0.888	0.8877	0.587	0.469	
Condition factor	1	2.171	2.1709	1.435	0.27	
Treatment*Challenge	1	0.246	0.2457	0.162	0.699	
Residuals	7	10.591	1.513			
**Cortisol receptor**	Df	SS	MS	F value	Pr(>F)	
Treatment	1	11.248	11.248	14.251	**0.00694**	**
Challenge	1	2.097	2.097	2.656	0.14716	
Condition factor	1	0.016	0.016	0.021	0.88933	
Treatment*Challenge	1	0.542	0.542	0.686	0.43479	
Residuals	7	5.525	0.789			
