# Supplementary material for: Parental investment matters for maternal and offspring immune defense in the mouthbrooding cichlid Astatotilapia burtoni
Source: BMC Evol Biol. 2017 Dec 20;17:264. doi: 10.1186/s12862-017-1109-6 (PMC5738712; doi:10.1186/s12862-017-1109-6)
Supplement: Supplementary file 3 — Tukey HSD test on candidate genes from brooding versus non-brooding females: Posthoc test following significant results from two-way ANCOVA. P-values marked in bold are in agreement with the results from the univariate analysis. (PDF 32 kb) [file 12862_2017_1109_MOESM3_ESM.pdf]

S3 Table: Tukey HSD test on candidate genes from brooding versus non-brooding females: Posthoc test following significant results from two-way ANCOVA. P-values marked in **bold** are in agreement with the results from the univariate analysis.

chemokine receptor

	control naive (C-)				control virbio (C+)				brooding naive (B-)			
	diff	lower	upper	p value	diff	lower	upper	p value	diff	lower	upper	p value
control naive (C-)												
control virbio (C+)	0.31	-1.63	2.24	0.9513								
brooding naive (B-)	0.67	-1.27	2.61	0.6779	0.97	-0.97	2.91	0.4068				
brooding vibrio (B+)	2.03	0.09	3.97	**0.0411**	1.72	-3.66	0.22	0.0807	2.70	0.76	4.64	**0.0102**

lectine

	control naive (C-)				control virbio (C+)				brooding naive (B-)			
	diff	lower	upper	p value	diff	lower	upper	p value	diff	lower	upper	p value
control naive (C-)												
control virbio (C+)	0.94	-1.21	3.09	0.5108								
brooding naive (B-)	0.56	-2.71	1.58	0.8223	0.38	-1.77	2.52	0.9336				
brooding vibrio (B+)	3.91	1.77	6.06	**0.0022**	2.97	-5.12	-0.83	**0.0105**	3.35	1.21	5.50	**0.0055**
