# Supplementary material for: Parental investment matters for maternal and offspring immune defense in the mouthbrooding cichlid Astatotilapia burtoni
Source: BMC Evol Biol. 2017 Dec 20;17:264. doi: 10.1186/s12862-017-1109-6 (PMC5738712; doi:10.1186/s12862-017-1109-6)
Supplement: Supplementary file 4 — Tukey HSD test on cellular immune parameter from males, reproducing and non-reproducing females: Posthoc test following significant results from two-way ANCOVA. P-values marked in bold are in agreement with the results from the univariate analysis. (PDF 32 kb) [file 12862_2017_1109_MOESM4_ESM.pdf]

S4 Table: Tukey HSD test on cellular immune parameter from males, reproducing and non-reproducing females: Posthoc test following significant results from two-way ANCOVA. P-values marked in bold are in agreement with the results from the univariate analysis.

Lymphocyte / Monocyte

	Male				Reproduction			
	diff	lower	upper	p value	diff	lower	upper	p value
Male								
Reproduction	1.45	-2.43	-0.48	**0.0031**				
Control	0.18	-1.20	0.85	0.9012	1.63	-2.57	-0.69	**0.0007**

Active / Inactive Cells

	Male				Reproduction			
	diff	lower	upper	p value	diff	lower	upper	p value
Male								
Reproduction	0.54	0.03	1.05	**0.0373**				
Control	0.09	-0.62	0.45	0.9140	0.452	-0.0379	0.9419	0.0738
