# Supplementary material for: Parental investment matters for maternal and offspring immune defense in the mouthbrooding cichlid Astatotilapia burtoni
Source: BMC Evol Biol. 2017 Dec 20;17:264. doi: 10.1186/s12862-017-1109-6 (PMC5738712; doi:10.1186/s12862-017-1109-6)
Supplement: Supplementary file 5 — ANCOVA on candidate genes from males, reproducing and non-reproducing females: Univariate analysis following significant gene groups in multivariate PERMANCOVA. Sigificant p-Values are marked with asterisk (code: p-Value >0.001 ***; > 0.01 **; > 0.01*). P-values marked additionally in bold are in agreement with the results from the multivariate analysis. (PDF 69 kb) [file 12862_2017_1109_MOESM5_ESM.pdf]

S5 Table: ANCOVA on candidate genes from males, reproducing and non reproducing females: Univariate analysis following significant gene groups in multivariate PERMANCOVA. Sigificant p-Values are marked with asterisk (code: p-Value > 0.001 ***; >0.01 **; >0.01*). Bold **p-values** are in agreement with the multivariate analysis

ANCOVA aov(x~treatment*challenge+K)

**Allograph inflammation factor**	Df	SS	MS	F value	Pr(>F)	
Treatment	2	1.052	0.526	1.163	0.335	
Challenge	1	0.181	0.181	0.4	0.535	
Condition factor	1	0.14	0.1396	0.309	0.585	
Treatment*Challenge	1	0.214	0.2139	0.473	0.5	
Residuals	18	8.138	0.4521			
**Androgen receptor A**	Df	SS	MS	F value	Pr(>F)	
Treatment	2	2.189	1.0947	2.947	0.0782	.
Challenge	1	1.092	1.0921	2.94	0.1036	
Condition factor	1	0.704	0.7038	1.894	0.1856	
Treatment*Challenge	1	0.005	0.0046	0.012	0.9128	
Residuals	18	6.687	0.3715			
**Androgen receptor B**	Df	SS	MS	F value	Pr(>F)	
Treatment	2	4.161	2.0803	8.789	**0.00217**	**
Challenge	1	0.65	0.6504	2.748	**0.11473**	
Condition factor	1	0	0	0	0.99323	
Treatment*Challenge	1	0.887	0.8872	3.748	0.06874	.
Residuals	18	4.261	0.2367			
**Aromatase B**	Df	SS	MS	F value	Pr(>F)	
Treatment	2	3.946	1.973	1.266	0.306	
Challenge	1	0.009	0.009	0.006	0.94	
Condition factor	1	0.981	0.981	0.629	0.438	
Treatment*Challenge	1	1.189	1.189	0.762	0.394	
Residuals	18	28.061	1.559			
**Complement component 1q**	Df	SS	MS	F value	Pr(>F)	
Treatment	2	1.13	0.5648	1.359	0.282	
Challenge	1	0.086	0.0863	0.207	0.654	
Condition factor	1	0.812	0.8119	1.953	0.179	
Treatment*Challenge	1	0.389	0.3892	0.936	0.346	
Residuals	18	7.484	0.4158			
**Complement component 9**	Df	SS	MS	F value	Pr(>F)	
Treatment	2	44.38	22.189	2.564	0.105	
Challenge	1	0.87	0.868	0.1	0.755	
Condition factor	1	3.31	3.312	0.383	0.544	
Treatment*Challenge	1	7.82	7.821	0.904	0.354	
Residuals	18	155.77	8.654			
**Calreticulin 1**	Df	SS	MS	F value	Pr(>F)	
Treatment	2	0.06	0.0298	0.098	0.907	
Challenge	1	0.014	0.0138	0.045	0.834	
Condition factor	1	0.595	0.5949	1.952	0.179	
Treatment*Challenge	1	0	0.0002	0.001	0.981	
Residuals	18	5.485	0.3047			
**Calreticulin 3**	Df	SS	MS	F value	Pr(>F)	
Treatment	2	109.77	54.88	10.73	**0.00085**	***
Challenge	1	0.39	0.39	0.077	0.78459	
Condition factor	1	1.98	1.98	0.387	0.54168	
Treatment*Challenge	1	3.87	3.87	0.757	0.39582	
Residuals	18	92.07	5.11			

**Catalase**	Df	SS	MS	F value	Pr(>F)	
Treatment	2	0.504	0.2521	0.54	0.592	
Challenge	1	0.024	0.0243	0.052	0.822	
Condition factor	1	0.003	0.0035	0.007	0.932	
Treatment*Challenge	1	0.41	0.4101	0.879	0.361	
Residuals	18	8.402	0.4668			
**Fibronectin beta antigen CD29**	Df	SS	MS	F value	Pr(>F)	
Treatment	2	51.51	25.753	3.769	**0.0429**	*
Challenge	1	0.05	0.055	0.008	0.9297	
Condition factor	1	10.28	10.276	1.504	0.2359	
Treatment*Challenge	1	10.71	10.712	1.568	0.2266	
Residuals	18	122.99	6.833			
**Fibronectin beta antigen CD81**	Df	SS	MS	F value	Pr(>F)	
Treatment	2	79.57	39.79	7.776	**0.00368**	**
Challenge	1	1.56	1.56	0.305	0.58735	
Condition factor	1	0.29	0.29	0.058	0.81304	
Treatment*Challenge	1	12.02	12.02	2.349	0.14277	
Residuals	18	92.1	5.12			
**Chemokine receptor 9**	Df	SS	MS	F value	Pr(>F)	
Treatment	2	2.79	1.396	0.764	0.48	
Challenge	1	0.51	0.5055	0.277	0.605	
Condition factor	1	0.1	0.0984	0.054	0.819	
Treatment*Challenge	1	0.04	0.0385	0.021	0.886	
Residuals	18	32.9	1.8277			
**Copper zink dismutase**	Df	SS	MS	F value	Pr(>F)	
Treatment	2	0.216	0.1081	0.311	0.737	
Challenge	1	0.024	0.0236	0.068	0.797	
Condition factor	1	0.492	0.4918	1.416	0.25	
Treatment*Challenge	1	0.077	0.0768	0.221	0.644	
Residuals	18	6.253	0.3474			
**Cortisol receptor**	Df	SS	MS	F value	Pr(>F)	
Treatment	2	10.31	5.156	0.779	0.474	
Challenge	1	0.12	0.116	0.017	0.896	
Condition factor	1	2.79	2.794	0.422	0.524	
Treatment*Challenge	1	2.22	2.223	0.336	0.569	
Residuals	18	119.2	6.622			
**DNA methyltransferase 1**	Df	SS	MS	F value	Pr(>F)	
Treatment	2	1.3573	0.6786	6.278	**0.00854**	**
Challenge	1	0.0865	0.0865	0.8	0.38283	
Condition factor	1	0.3223	0.3223	2.982	0.10132	
Treatment*Challenge	1	0.1535	0.1535	1.42	0.24881	
Residuals	18	1.9456	0.1081			
**Elongation Factor 1**	Df	SS	MS	F value	Pr(>F)	
Treatment	2	129.95	64.98	4.231	**0.0312**	*
Challenge	1	2.15	2.15	0.14	0.7124	
Condition factor	1	0.56	0.56	0.037	0.8501	
Treatment*Challenge	1	5.28	5.28	0.344	0.5649	
Residuals	18	276.44	15.36			
**Early growth response protein 1**	Df	SS	MS	F value	Pr(>F)	
Treatment	2	2.483	1.2414	0.855	0.442	
Challenge	1	0.028	0.0279	0.019	0.891	
Condition factor	1	0.486	0.4863	0.335	0.57	
Treatment*Challenge	1	0.126	0.1257	0.087	0.772	

Residuals	18	26.128	1.4516			
**Histone lysine methyltransferase**	Df	SS	MS	F value	Pr(>F)	
Treatment	2	3.14	1.569	0.445	0.6474	
Challenge	1	2.81	2.811	0.798	0.3835	
Condition factor	1	0.22	0.222	0.063	0.8045	
Treatment*Challenge	1	11.12	11.118	3.156	0.0926	.
Residuals	18	63.41	3.523			
**Coagulation factor II / Thrombin**	Df	SS	MS	F value	Pr(>F)	
Treatment	2	16.08	8.04	3.919	**0.0387**	*
Challenge	1	0.1	0.098	0.048	0.8291	
Condition factor	1	0.27	0.272	0.132	0.7202	
Treatment*Challenge	1	0.43	0.429	0.209	0.6531	
Residuals	18	36.93	2.052			
**FAM60 A Protein**	Df	SS	MS	F value	Pr(>F)	
Treatment	2	1.317	0.6583	2.434	0.116	
Challenge	1	0.156	0.1563	0.578	0.457	
Condition factor	1	0.21	0.2102	0.777	0.39	
Treatment*Challenge	1	0.035	0.0351	0.13	0.723	
Residuals	18	4.869	0.2705			
**Fc fragment of IgL**	Df	SS	MS	F value	Pr(>F)	
Treatment	2	6.99	3.496	0.815	0.4584	
Challenge	1	1.56	1.564	0.365	0.5535	
Condition factor	1	17.34	17.34	4.042	0.0596	.
Treatment*Challenge	1	18.09	18.086	4.215	0.0549	.
Residuals	18	77.23	4.29			
**Growth hormone releasing hormone**	Df	SS	MS	F value	Pr(>F)	
Treatment	2	34.09	17.047	2.36	0.123	
Challenge	1	0.76	0.762	0.105	0.749	
Condition factor	1	0.07	0.074	0.01	0.92	
Treatment*Challenge	1	19.11	19.108	2.645	0.121	
Residuals	18	130.04	7.225			
**Trypsin I**	Df	SS	MS	F value	Pr(>F)	
Treatment	2	58.49	29.246	5.323	**0.0153**	*
Challenge	1	0.35	0.347	0.063	0.8044	
Condition factor	1	4.1	4.1	0.746	0.399	
Treatment*Challenge	1	7.41	7.411	1.349	0.2606	
Residuals	18	98.89	5.494			
**Histone deacetylase**	Df	SS	MS	F value	Pr(>F)	
Treatment	2	2.83	1.414	0.312	0.736	
Challenge	1	0.16	0.157	0.035	0.854	
Condition factor	1	3.06	3.056	0.675	0.422	
Treatment*Challenge	1	0.49	0.494	0.109	0.745	
Residuals	18	81.54	4.53			
**Histone demethylase**	Df	SS	MS	F value	Pr(>F)	
Treatment	2	1.371	0.6855	2.985	0.076	.
Challenge	1	0.199	0.199	0.866	0.364	
Condition factor	1	0.6	0.6	2.612	0.123	
Treatment*Challenge	1	0.468	0.4678	2.036	0.171	
Residuals	18	4.134	0.2297			
**Hepcidin**	Df	SS	MS	F value	Pr(>F)	
Treatment	2	80.28	40.14	6.478	**0.0076**	**
Challenge	1	0.2	0.2	0.032	0.8596	
Condition factor	1	11.11	11.11	1.793	0.1973	

Treatment*Challenge	1	8	8	1.291	0.2708	
Residuals	18	111.54	6.2			
**Major histocompatibility complex II beta**	Df	SS	MS	F value	Pr(>F)	
Treatment	2	264.3	132.15	10.73	**0.00085**	***
Challenge	1	5.6	5.6	0.455	0.50852	
Condition factor	1	2.56	2.56	0.208	0.65394	
Treatment*Challenge	1	14.75	14.75	1.197	0.28828	
Residuals	18	221.69	12.32			
**Heat shock protein 60**	Df	SS	MS	F value	Pr(>F)	
Treatment	2	1.096	0.5482	1.551	0.239	
Challenge	1	0.04	0.0402	0.114	0.74	
Condition factor	1	0.032	0.0317	0.09	0.768	
Treatment*Challenge	1	0.634	0.6339	1.793	0.197	
Residuals	18	6.362	0.3535			
**Heat shock protein 70**	Df	SS	MS	F value	Pr(>F)	
Treatment	2	40.01	20.004	5.157	**0.017**	*
Challenge	1	1.71	1.709	0.441	0.515	
Condition factor	1	4.7	4.696	1.211	0.286	
Treatment*Challenge	1	2.37	2.375	0.612	0.444	
Residuals	18	69.82	3.879			
**Heat shock protein 90**	Df	SS	MS	F value	Pr(>F)	
Treatment	2	1.76	0.879	0.15	0.862	
Challenge	1	0.05	0.048	0.008	0.929	
Condition factor	1	11.7	11.702	1.992	0.175	
Treatment*Challenge	1	3.76	3.756	0.639	0.434	
Residuals	18	105.77	5.876			
**Immunoglobulin L light chain**	Df	SS	MS	F value	Pr(>F)	
Treatment	2	0.118	0.0591	0.039	0.962	
Challenge	1	0.016	0.0158	0.01	0.92	
Condition factor	1	0.092	0.0918	0.061	0.808	
Treatment*Challenge	1	1.026	1.0258	0.682	0.42	
Residuals	18	27.086	1.5048			
**Interleukin 10**	Df	SS	MS	F value	Pr(>F)	
Treatment	2	2.575	1.2877	3.987	**0.0369**	*
Challenge	1	0.302	0.3016	0.934	0.3467	
Condition factor	1	0.21	0.2101	0.65	0.4305	
Treatment*Challenge	1	0.03	0.0302	0.094	0.7632	
Residuals	18	5.813	0.323			
**Integrin alpha 2**	Df	SS	MS	F value	Pr(>F)	
Treatment	2	0.989	0.4947	1.74	0.204	
Challenge	1	0.013	0.0133	0.047	0.831	
Condition factor	1	0.107	0.1072	0.377	0.547	
Treatment*Challenge	1	0.002	0.0018	0.006	0.937	
Residuals	18	5.118	0.2843			
**Latescidin 2**	Df	SS	MS	F value	Pr(>F)	
Treatment	2	89.48	44.74	6.688	**0.00673**	**
Challenge	1	3.12	3.12	0.467	0.50325	
Condition factor	1	12.16	12.16	1.818	0.19424	
Treatment*Challenge	1	11.51	11.51	1.72	0.20615	
Residuals	18	120.4	6.69			
**Lymphocyte cytosolic factor I**	Df	SS	MS	F value	Pr(>F)	
Treatment	2	0.86	0.4298	1.088	0.358	
Challenge	1	0.052	0.0515	0.13	0.722	

Condition factor	1	0.051	0.0512	0.13	0.723	
Treatment*Challenge	1	0.03	0.0296	0.075	0.787	
Residuals	18	7.113	0.3952			
**Lectine**	Df	SS	MS	F value	Pr(>F)	
Treatment	2	12.51	6.253	0.617	0.551	
Challenge	1	0.61	0.609	0.06	0.809	
Condition factor	1	1.23	1.23	0.121	0.732	
Treatment*Challenge	1	0.15	0.154	0.015	0.903	
Residuals	18	182.52	10.14			
**Lysine specific demethylase**	Df	SS	MS	F value	Pr(>F)	
Treatment	2	2.568	1.284	2.226	0.137	
Challenge	1	0.043	0.0429	0.074	0.788	
Condition factor	1	0.181	0.1813	0.314	0.582	
Treatment*Challenge	1	0.163	0.163	0.283	0.601	
Residuals	18	10.381	0.5767			
**Major histocompatibility complex I antigen F10**	Df	SS	MS	F value	Pr(>F)	
Treatment	2	8.39	4.2	0.258	0.7752	
Challenge	1	10.59	10.59	0.651	0.4301	
Condition factor	1	48.35	48.35	2.976	0.1016	
Treatment*Challenge	1	75.57	75.57	4.651	0.0448	*
Residuals	18	292.48	16.25			
**Myogenic regulatory factors**	Df	SS	MS	F value	Pr(>F)	
Treatment	2	61.99	30.993	12.838	**0.00034**	***
Challenge	1	0.07	0.068	0.028	0.86889	
Condition factor	1	0.36	0.357	0.148	0.70489	
Treatment*Challenge	1	3.96	3.958	1.639	0.21664	
Residuals	18	43.45	2.414			
**Opsin 1**	Df	SS	MS	F value	Pr(>F)	
Treatment	2	85.64	42.82	8.446	**0.00259**	**
Challenge	1	1.52	1.52	0.299	0.59097	
Condition factor	1	0.05	0.05	0.009	0.92425	
Treatment*Challenge	1	1.97	1.97	0.389	0.5407	
Residuals	18	91.26	5.07			
**Proprotein convertase subtilisin**	Df	SS	MS	F value	Pr(>F)	
Treatment	2	1.383	0.6917	2.648	0.0981	.
Challenge	1	0.158	0.1583	0.606	0.4463	
Condition factor	1	0.001	0.0007	0.003	0.9584	
Treatment*Challenge	1	0.041	0.0413	0.158	0.6955	
Residuals	18	4.701	0.2612			
**Pentraxin 4**	Df	SS	MS	F value	Pr(>F)	
Treatment	2	10.88	5.442	0.955	0.4035	
Challenge	1	0.07	0.073	0.013	0.911	
Condition factor	1	0.81	0.813	0.143	0.7101	
Treatment*Challenge	1	20.31	20.315	3.565	0.0752	.
Residuals	18	102.57	5.699			
**Ribosomal protein A3**	Df	SS	MS	F value	Pr(>F)	
Treatment	2	2.201	1.1005	3.343	0.0583	.
Challenge	1	0.002	0.0016	0.005	0.9449	
Condition factor	1	0.216	0.2161	0.656	0.4284	
Treatment*Challenge	1	0.105	0.1047	0.318	0.5797	
Residuals	18	5.926	0.3292			
**Serum amyloid A**	Df	SS	MS	F value	Pr(>F)	
Treatment	2	52.6	26.301	3.87	**0.04**	*

Challenge	1	4.43	4.428	0.652	0.43	
Condition factor	1	2.97	2.969	0.437	0.517	
Treatment*Challenge	1	1.7	1.698	0.25	0.623	
Residuals	18	122.34	6.797			
**Tumor necrose factor beta**	Df	SS	MS	F value	Pr(>F)	
Treatment	2	2.589	1.2944	2.867	0.083	.
Challenge	1	0.247	0.2471	0.547	0.469	
Condition factor	1	0.294	0.2937	0.651	0.43	
Treatment*Challenge	1	0.109	0.1088	0.241	0.629	
Residuals	18	8.126	0.4514			
