# Supplementary material for: Parental investment matters for maternal and offspring immune defense in the mouthbrooding cichlid Astatotilapia burtoni
Source: BMC Evol Biol. 2017 Dec 20;17:264. doi: 10.1186/s12862-017-1109-6 (PMC5738712; doi:10.1186/s12862-017-1109-6)
Supplement: Supplementary file 6 — Tukey HSD test on candidate genes from males, reproducing and non-reproducing females: Posthoc test following significant results from two-way ANCOVA. P-values marked in bold are in agreement with the results from the univariate analysis. (PDF 41 kb) [file 12862_2017_1109_MOESM6_ESM.pdf]

S6 Table: Tukey HSD test on candidate genes from males, reproducing and non-reproducing females: Posthoc test following significant results from two-way ANCOVA. P-values marked in bold are in agreement with the results from the univariate analysis.

MHC II b

	Male				Reproduction			
	diff	lower	upper	p value	diff	lower	upper	p value
Male								
Reproduction	8.01	-12.35	-3.67	**0.0004**				
Control	4.80	0.09	9.51	**0.0452**	3.21	-7.55	1.13	0.1721

Il10

	Male				Reproduction			
	diff	lower	upper	p value	diff	lower	upper	p value
Male								
Reproduction	1.45	-2.43	-0.48	**0.0031**				
Control	0.78	-1.48	-0.07	**0.0288**	0.18	-0.88	0.52	0.7920

CD8

	Male				Reproduction			
	diff	lower	upper	p value	diff	lower	upper	p value
Male								
Reproduction	0.78	0.04	1.53	**0.0373**				
Control	0.08	-0.73	0.88	0.9666	0.86	0.12	1.61	**0.0212**

calreticulin 3

	Male				Reproduction			
	diff	lower	upper	p value	diff	lower	upper	p value
Male								
Reproduction	5.16	-7.97	-2.35	**0.0005**				
Control	3.11	0.07	6.16	**0.0446**	2.05	-4.86	0.76	0.1800

Serum Amyloid

	Male				Reproduction			
	diff	lower	upper	p value	diff	lower	upper	p value
Male								
Reproduction	3.25	0.05	6.45	**0.0458**				
Control	0.57	-4.03	2.90	0.9104	2.69	-0.51	5.89	0.1093

thrombin receptor

	Male				Reproduction			
	diff	lower	upper	p value	diff	lower	upper	p value
Male								
Reproduction	1.97	-3.72	-0.22	**0.0256**				
Control	1.03	-0.86	2.93	0.3674	0.94	-2.68	0.81	0.3804

trypsin

	Male				Reproduction			
	diff	lower	upper	p value	diff	lower	upper	p value
Male								
Reproduction	3.00	0.12	5.87	**0.0405**				
Control	0.32	-2.80	3.44	0.9628	3.32	0.44	6.20	**0.0223**

latescidin 2

	Male				Reproduction			
	diff	lower	upper	p value	diff	lower	upper	p value
Male								
Reproduction	3.86	0.63	7.08	**0.0179**				
Control	0.12	-3.38	3.62	0.9958	3.98	0.75	7.20	**0.0146**

hepcidine

	Male				Reproduction			
	diff	lower	upper	p value	diff	lower	upper	p value
Male								
Reproduction	3.93	0.82	7.04	**0.0123**				
Control	0.48	-3.86	2.90	0.9312	3.45	0.34	6.57	**0.0284**

HSP70

	Male				Reproduction			
	diff	lower	upper	p value	diff	lower	upper	p value
Male								
Reproduction	0.81	0.17	1.44	**0.0116**				
Control	0.71	-1.40	-0.02	**0.0418**	0.10	-0.54	0.73	0.9222

elongation factor

	Male				Reproduction			
	diff	lower	upper	p value	diff	lower	upper	p value
Male								
Reproduction	5.49	-10.28	-0.70	**0.0231**				
Control	2.16	-3.03	7.36	0.5514	3.33	-8.12	1.46	0.2074

opsin

	Male				Reproduction			
	diff	lower	upper	p value	diff	lower	upper	p value
Male								
Reproduction	3.64	0.89	6.38	**0.0087**				
Control	0.37	-2.61	3.35	0.9470	4.01	1.26	6.75	**0.0041**

myogenic regulation

	Male				Reproduction			
	diff	lower	upper	p value	diff	lower	upper	p value
Male								
Reproduction	3.41	1.51	5.30	**0.0006**				
Control	0.31	-2.37	1.74	0.9202	3.09	1.20	4.99	**0.0015**

methyltransferase

	Male				Reproduction			
	diff	lower	upper	p value	diff	lower	upper	p value
Male								
Reproduction	0.57	0.13	1.02	**0.0101**				
Control	0.34	-0.82	0.14	0.1917	0.34	-0.82	0.14	0.1917

androgen receptor B

	Male				Reproduction			
	diff	lower	upper	p value	diff	lower	upper	p value
Male								
Reproduction	0.83	0.23	1.43	**0.0061**				
Control	0.03	-0.62	0.68	0.9917	0.86	0.26	1.46	**0.0046**
