# Supplementary material for: Parental investment matters for maternal and offspring immune defense in the mouthbrooding cichlid Astatotilapia burtoni
Source: BMC Evol Biol. 2017 Dec 20;17:264. doi: 10.1186/s12862-017-1109-6 (PMC5738712; doi:10.1186/s12862-017-1109-6)
Supplement: Supplementary file 7 — nested ANOVA on candidate genes from mouthbred juveniles: Univariate analysis following significant gene groups in multivariate nested MANOVA. Significant p-Values are marked with asterisk (code: p-Value > 0.001 ***; > 0.01 **; > 0.01*). P-values marked additionally in bold are in agreement with the results from the multivariate analysis. (PDF 74 kb) [file 12862_2017_1109_MOESM7_ESM.pdf]

S7 Table: nested ANOVA on candidate genes from mouthbred juveniles: Univariate analysis following significant gene groups in multivariate nested MANOVA. Sigificant p-Values are marked with asterisk (code: p-Value > 0.001 ***; >0.01 **; >0.01*). Bold **p-values** are in agreement with the multivariate analysis

nested ANOVA aov(x~jtreatment*ftreatment+ftreatment%in%family)

**Allograph inflammation factor**	Df	SS	MS	F value	Pr(>F)	
juvenile challenge	2	2.64E-07	1.32E-07	0.9166	0.4059	
maternal treatment	1	8.83E-08	8.83E-08	0.6137	0.4368	
juvenile challenge * maternal treatment	2	3.65E-07	1.83E-07	1.2681	0.2895	
maternal treatment in famliy	2	3.08E-07	1.54E-07	1.0685	0.3505	
Residuals	55	7.92E-06	1.44E-07			
**Androgen receptor A**	Df	SS	MS	F value	Pr(>F)	
juvenile challenge	2	3.59	1.7972	0.3044	0.73883	
maternal treatment	1	18.47	18.4743	3.1286	0.08248	.
juvenile challenge * maternal treatment	2	23.19	11.5933	1.9633	0.15011	
maternal treatment in famliy	2	23.13	11.5647	1.9584	0.15079	
Residuals	55	324.78	5.905			
**Androgen receptor B**	Df	SS	MS	F value	Pr(>F)	
juvenile challenge	2	3064	1532	0.2629	0.769779	
maternal treatment	1	33837	33837	5.8062	**0.019344**	*
juvenile challenge * maternal treatment	2	8162	4081	0.7003	0.500806	
maternal treatment in famliy	2	62023	31011	5.3214	0.007715	**
Residuals	55	320524	5828			
**Aromatase B**	Df	SS	MS	F value	Pr(>F)	
juvenile challenge	2	4.75E-07	2.38E-07	3.6486	**0.03252**	*
maternal treatment	1	3.89E-07	3.89E-07	5.9754	**0.01774**	*
juvenile challenge * maternal treatment	2	5.03E-07	2.51E-07	3.8595	0.02701	*
maternal treatment in famliy	2	2.86E-07	1.43E-07	2.1977	0.12072	
Residuals	55	3.58E-06	6.51E-08			
**Complement component 1q**	Df	SS	MS	F value	Pr(>F)	
juvenile challenge	2	1.25E-06	6.25E-07	3.5572	**0.035253**	*
maternal treatment	1	3.59E-07	3.59E-07	2.0396	0.158906	
juvenile challenge * maternal treatment	2	2.40E-06	1.20E-06	6.8183	**0.002263**	**
maternal treatment in famliy	2	2.48E-07	1.24E-07	0.7052	0.49842	
Residuals	55	9.67E-06	1.76E-07			
**Complement component 9**	Df	SS	MS	F value	Pr(>F)	
juvenile challenge	2	1.043	0.52149	0.9829	0.38072	
maternal treatment	1	0.7514	0.75138	1.4161	0.23915	
juvenile challenge * maternal treatment	2	1.3599	0.67994	1.2815	0.28578	
maternal treatment in famliy	2	3.8644	1.93221	3.6417	0.03272	*
Residuals	55	29.1822	0.53059			
**Calreticulin 1**	Df	SS	MS	F value	Pr(>F)	
juvenile challenge	2	8.06E-07	4.03E-07	3.4109	**0.04014**	*
maternal treatment	1	7.23E-08	7.23E-08	0.6117	0.43751	
juvenile challenge * maternal treatment	2	9.71E-07	4.85E-07	4.1078	0.02174	*

maternal treatment in famliy	2	5.76E-07	2.88E-07	2.4391	0.09662	.
Residuals	55	6.50E-06	1.18E-07			
**Calreticulin 3**	Df	SS	MS	F value	Pr(>F)	
juvenile challenge	2	1.89E-06	9.45E-07	3.0182	0.057051	.
maternal treatment	1	1.07E-07	1.07E-07	0.3416	0.561296	
juvenile challenge * maternal treatment	2	4.13E-06	2.06E-06	6.5933	0.002712	**
maternal treatment in famliy	2	1.15E-06	5.75E-07	1.8374	0.168872	
Residuals	55	1.72E-05	3.13E-07			
**Catalase**	Df	SS	MS	F value	Pr(>F)	
juvenile challenge	2	1.57E-07	7.84E-08	1.0475	0.357713	
maternal treatment	1	1.94E-07	1.94E-07	2.5963	0.112839	
juvenile challenge * maternal treatment	2	7.62E-07	3.81E-07	5.0893	0.009377	**
maternal treatment in famliy	2	3.45E-07	1.72E-07	2.3019	0.109634	
Residuals	55	4.12E-06	7.49E-08			
**Fibronectin beta antigen CD29**	Df	SS	MS	F value	Pr(>F)	
juvenile challenge	2	39658	19829	0.8329	0.4402	
maternal treatment	1	6893	6893	0.2895	0.5927	
juvenile challenge * maternal treatment	2	10940	5470	0.2298	0.7955	
maternal treatment in famliy	2	73463	36732	1.5429	0.2229	
Residuals	55	1309356	23806			
**Fibronectin beta antigen CD81**	Df	SS	MS	F value	Pr(>F)	
juvenile challenge	2	7.99E-07	3.99E-07	2.9752	0.059308	.
maternal treatment	1	1.58E-07	1.58E-07	1.1761	0.282874	
juvenile challenge * maternal treatment	2	1.75E-06	8.74E-07	6.5106	0.002899	**
maternal treatment in famliy	2	3.87E-08	1.94E-08	0.1442	0.866	
Residuals	55	7.38E-06	1.34E-07			
**Chemokine receptor 9**	Df	SS	MS	F value	Pr(>F)	
juvenile challenge	2	1.98E-07	9.88E-08	1.3158	0.276583	
maternal treatment	1	7.96E-07	7.96E-07	10.6012	**0.001937**	**
juvenile challenge * maternal treatment	2	6.43E-07	3.21E-07	4.281	0.018708	*
maternal treatment in famliy	2	1.90E-07	9.47E-08	1.2618	0.291203	
Residuals	55	4.13E-06	7.51E-08			
**Copper zink dismutase**						
juvenile challenge	2	1.09E-06	5.45E-07	1.0776	0.3475	
maternal treatment	1	7.20E-07	7.20E-07	1.4226	0.23809	
juvenile challenge * maternal treatment	2	3.73E-06	1.87E-06	3.6897	0.03136	*
maternal treatment in famliy	2	3.86E-06	1.93E-06	3.8108	0.02819	*
Residuals	55	2.78E-05	5.06E-07			
**Cortisol receptor**	Df	SS	MS	F value	Pr(>F)	
juvenile challenge	2	843	421.7	0.5703	0.568655	
maternal treatment	1	142	141.7	0.1916	0.663266	
juvenile challenge * maternal treatment	2	7443	3721.7	5.0329	0.009835	**
maternal treatment in famliy	2	265	132.3	0.1789	0.836687	
Residuals	55	40671	739.5			
**DNA methyltransferase 1**	Df	SS	MS	F value	Pr(>F)	
juvenile challenge	2	8.17E-07	4.08E-07	3.5972	0.03403	*

maternal treatment	1	1.45E-07	1.45E-07	1.2772	0.26333	
juvenile challenge * maternal treatment	2	5.01E-07	2.50E-07	2.2042	0.11999	
maternal treatment in famliy	2	3.88E-07	1.94E-07	1.7106	0.19024	
Residuals	55	6.24E-06	1.14E-07			
**Elongation Factor 1**	Df	SS	MS	F value	Pr(>F)	
juvenile challenge	2	4.96E-06	2.48E-06	1.9312	0.1547	
maternal treatment	1	1.21E-06	1.21E-06	0.9433	0.3357	
juvenile challenge * maternal treatment	2	4.51E-06	2.25E-06	1.7535	0.1827	
maternal treatment in famliy	2	7.64E-07	3.82E-07	0.2974	0.7439	
Residuals	55	7.07E-05	1.28E-06			
**Early growth response protein 1**	Df	SS	MS	F value	Pr(>F)	
juvenile challenge	2	1.11E-06	5.55E-07	5.8303	**0.005053**	**
maternal treatment	1	5.14E-07	5.14E-07	5.4019	**0.023836**	*
juvenile challenge * maternal treatment	2	7.61E-07	3.81E-07	4.0009	0.023865	*
maternal treatment in famliy	2	6.92E-08	3.46E-08	0.3638	0.696697	
Residuals	55	5.23E-06	9.51E-08			
**Euchromatic histone lysine methyltransferase**						
juvenile challenge	2	345	173	0.0339	0.9666987	
maternal treatment	1	60038	60038	11.7949	**0.0011364**	**
juvenile challenge * maternal treatment	2	30874	15437	3.0327	0.0563113	.
maternal treatment in famliy	2	92088	46044	9.0456	0.0004015	***
Residuals	55	279960	5090			
**Coagulation factor II / Thrombin**	Df	SS	MS	F value	Pr(>F)	
juvenile challenge	2	0.0002352	1.18E-04	1.0439	0.3589	
maternal treatment	1	0.0002173	2.17E-04	1.929	0.1705	
juvenile challenge * maternal treatment	2	0.00022	1.10E-04	0.9763	0.3831	
maternal treatment in famliy	2	0.0000556	2.78E-05	0.2468	0.7822	
Residuals	55	0.0061971	1.13E-04			
**FAM60 A Protein**	Df	SS	MS	F value	Pr(>F)	
juvenile challenge	2	7.64E-06	3.82E-06	2.1676	0.12413	
maternal treatment	1	1.05E-05	1.05E-05	5.9375	0.01809	*
juvenile challenge * maternal treatment	2	3.72E-07	1.86E-07	0.1056	0.90001	
maternal treatment in famliy	2	1.32E-05	6.60E-06	3.7471	0.02981	*
Residuals	55	9.69E-05	1.76E-06			
**Fc fragment of IgL**	Df	SS	MS	F value	Pr(>F)	
juvenile challenge	2	119755	59878	3.4454	0.038926	*
maternal treatment	1	40687	40687	2.3412	0.131725	
juvenile challenge * maternal treatment	2	60996	30498	1.7549	0.18247	
maternal treatment in famliy	2	284040	142020	8.172	0.000781	***
Residuals	55	955836	17379			
**Growth hormone releasing hormone**	Df	SS	MS	F value	Pr(>F)	
juvenile challenge	2	846	423.1	0.0967	0.908	
maternal treatment	1	1127	1126.8	0.2575	0.6139	
juvenile challenge * maternal treatment	2	6027	3013.3	0.6887	0.5065	
maternal treatment in famliy	2	17113	8556.3	1.9555	0.1512	
Residuals	55	240657	4375.6			

**Trypsin I**	Df	SS	MS	F value	Pr(>F)	
juvenile challenge	2	31182	15591	0.6033	0.5506	
maternal treatment	1	10737	10737	0.4155	0.5219	
juvenile challenge * maternal treatment	2	40714	20357	0.7877	0.46	
maternal treatment in famliy	2	106658	53329	2.0635	0.1367	
Residuals	55	1421418	25844			
**Histone deacetylase**	Df	SS	MS	F value	Pr(>F)	
juvenile challenge	2	8.01E-07	4.00E-07	1.2218	0.3026	
maternal treatment	1	2.50E-09	2.51E-09	0.0077	0.9306	
juvenile challenge * maternal treatment	2	1.98E-07	9.90E-08	0.3021	0.7405	
maternal treatment in famliy	2	4.08E-07	2.04E-07	0.623	0.5401	
Residuals	55	1.80E-05	3.28E-07			
**Histone demethylase**	Df	SS	MS	F value	Pr(>F)	
juvenile challenge	2	1.82E-07	9.10E-08	3.646	0.03259	*
maternal treatment	1	2.26E-08	2.26E-08	0.9064	0.34525	
juvenile challenge * maternal treatment	2	6.15E-08	3.07E-08	1.2316	0.29973	
maternal treatment in famliy	2	1.80E-07	9.00E-08	3.6069	0.03374	*
Residuals	55	1.37E-06	2.49E-08			
**Hepcidin**	Df	SS	MS	F value	Pr(>F)	
juvenile challenge	2	11.794	5.897	3.4361	0.03925	*
maternal treatment	1	0.973	0.9727	0.5668	0.45476	
juvenile challenge * maternal treatment	2	0.734	0.3671	0.2139	0.80809	
maternal treatment in famliy	2	15.733	7.8667	4.5838	0.01441	*
Residuals	55	94.391	1.7162			
**Major histocompatibility complex II beta**	Df	SS	MS	F value	Pr(>F)	
juvenile challenge	2	2.65E-05	1.33E-05	2.5804	0.08489	.
maternal treatment	1	8.93E-07	8.93E-07	0.1738	0.67835	
juvenile challenge * maternal treatment	2	1.01E-05	5.04E-06	0.9825	0.38085	
maternal treatment in famliy	2	7.05E-06	3.52E-06	0.6861	0.5078	
Residuals	55	2.82E-04	5.13E-06			
**Heat shock protein 60**						
juvenile challenge	2	0.00001417	7.09E-06	0.9609	0.3889	
maternal treatment	1	0.0000002	1.99E-07	0.027	0.87	
juvenile challenge * maternal treatment	2	0.00000883	4.41E-06	0.5986	0.5531	
maternal treatment in famliy	2	0.00000115	5.75E-07	0.078	0.925	
Residuals	55	0.00040561	7.37E-06			
**Heat shock protein 70**	Df	SS	MS	F value	Pr(>F)	
juvenile challenge	2	1.20E-07	6.02E-08	1.1526	0.3233	
maternal treatment	1	3.98E-08	3.98E-08	0.7614	0.3867	
juvenile challenge * maternal treatment	2	1.33E-07	6.64E-08	1.2714	0.2885	
maternal treatment in famliy	2	1.77E-07	8.86E-08	1.6962	0.1928	
Residuals	55	2.87E-06	5.22E-08			
**Heat shock protein 90**	Df	SS	MS	F value	Pr(>F)	
juvenile challenge	2	2.171	1.08536	1.1168	0.3346	
maternal treatment	1	2.12	2.12009	2.1815	0.1454	
juvenile challenge * maternal treatment	2	1.908	0.95387	0.9815	0.3812	

maternal treatment in famliy	2	1.224	0.61182	0.6295	0.5366	
Residuals	55	53.452	0.97185			
**Immunoglobulin light chain**	Df	SS	MS	F value	Pr(>F)	
juvenile challenge	2	2.71E-07	1.36E-07	1.6262	0.2059795	
maternal treatment	1	1.60E-06	1.60E-06	19.2101	**5.32E-05**	***
juvenile challenge * maternal treatment	2	4.00E-07	2.00E-07	2.3942	0.1006957	
maternal treatment in famliy	2	1.31E-06	6.56E-07	7.8573	0.0009965	***
Residuals	55	4.59E-06	8.34E-08			
**Interleukin 10**	Df	SS	MS	F value	Pr(>F)	
juvenile challenge	2	1031	516	0.0737	0.929074	
maternal treatment	1	35458	35458	5.0665	**0.028409**	*
juvenile challenge * maternal treatment	2	823	412	0.0588	0.942952	
maternal treatment in famliy	2	75507	37754	5.3945	0.007257	**
Residuals	55	384920	6999			
**Integrin alpha 2**	Df	SS	MS	F value	Pr(>F)	
juvenile challenge	2	5.96E-07	2.98E-07	3.8214	0.02793	*
maternal treatment	1	3.14E-07	3.14E-07	4.0231	**0.04981**	*
juvenile challenge * maternal treatment	2	3.73E-07	1.87E-07	2.3949	0.10063	
maternal treatment in famliy	2	7.17E-08	3.58E-08	0.4598	0.63382	
Residuals	55	4.29E-06	7.80E-08			
**Latescidin 2**	Df	SS	MS	F value	Pr(>F)	
juvenile challenge	2	3.977	1.98865	2.1419	0.1271	
maternal treatment	1	0.029	0.0294	0.0317	0.8594	
juvenile challenge * maternal treatment	2	0.223	0.11174	0.1204	0.8868	
maternal treatment in famliy	2	2.68	1.34008	1.4434	0.2449	
Residuals	55	51.064	0.92844			
**Lymphocyte cytosolic factor I**	Df	SS	MS	F value	Pr(>F)	
juvenile challenge	2	6.43E-07	3.22E-07	3.1745	0.04957	*
maternal treatment	1	2.00E-10	2.00E-10	0.0019	0.96499	
juvenile challenge * maternal treatment	2	6.27E-07	3.13E-07	3.0921	0.05338	.
maternal treatment in famliy	2	3.50E-07	1.75E-07	1.727	0.18732	
Residuals	55	5.57E-06	1.01E-07			
**Lectine**	Df	SS	MS	F value	Pr(>F)	
juvenile challenge	2	0.0645	0.03225	1.2568	0.2926	
maternal treatment	1	0.02084	0.020838	0.8121	0.3714	
juvenile challenge * maternal treatment	2	0.05285	0.026424	1.0298	0.3639	
maternal treatment in famliy	2	0.00569	0.002845	0.1109	0.8953	
Residuals	55	1.4113	0.02566			
**Lysine specific demethylase**	Df	SS	MS	F value	Pr(>F)	
juvenile challenge	2	1.51E-06	7.55E-07	4.5929	0.0143	*
maternal treatment	1	6.81E-07	6.81E-07	4.142	**0.04666**	*
juvenile challenge * maternal treatment	2	1.14E-06	5.68E-07	3.4515	**0.03872**	*
maternal treatment in famliy	2	3.88E-07	1.94E-07	1.1783	0.31546	
Residuals	55	9.05E-06	1.64E-07			
**Major histocompatibility complex I antigen F10**	Df	SS	MS	F value	Pr(>F)	
juvenile challenge	2	0.002835	0.0014175	0.5325	0.59013	

maternal treatment	1	0.009762	0.0097616	3.667	0.06071	.
juvenile challenge * maternal treatment	2	0.004441	0.0022203	0.8341	0.4397	
maternal treatment in famliy	2	0.018393	0.0091963	3.4546	0.03861	*
Residuals	55	0.146412	0.002662			
**Myogenic regulatory factors**	Df	SS	MS	F value	Pr(>F)	
juvenile challenge	2	0.0001453	0.00007266	0.869	0.42504	
maternal treatment	1	0.0003696	0.00036964	4.4208	**0.04009**	*
juvenile challenge * maternal treatment	2	0.0003167	0.00015837	1.894	0.16015	
maternal treatment in famliy	2	0.0001833	0.00009165	1.0961	0.34137	
Residuals	55	0.0045988	0.00008362			
**Opsin 1**	Df	SS	MS	F value	Pr(>F)	
juvenile challenge	2	0.0002315	1.16E-04	1.959	0.15071	
maternal treatment	1	0.0000022	2.17E-06	0.0367	0.84871	
juvenile challenge * maternal treatment	2	0.00002	1.00E-05	0.1696	0.84442	
maternal treatment in famliy	2	0.00029	1.45E-04	2.4547	0.09525	.
Residuals	55	0.0032494	5.91E-05			
**Proprotein convertase subtilisin**	Df	SS	MS	F value	Pr(>F)	
juvenile challenge	2	1.94E-07	9.72E-08	0.8877	0.4174	
maternal treatment	1	2.35E-07	2.35E-07	2.1443	0.1488	
juvenile challenge * maternal treatment	2	1.65E-07	8.25E-08	0.7539	0.4753	
maternal treatment in famliy	2	1.91E-07	9.56E-08	0.8733	0.4233	
Residuals	55	6.02E-06	1.09E-07			
**Pentraxin 4**	Df	SS	MS	F value	Pr(>F)	
juvenile challenge	2	24494	12247	1.31	0.2781153	
maternal treatment	1	40270	40270	4.3074	**0.0426355**	*
juvenile challenge * maternal treatment	2	20868	10434	1.1161	0.3348692	
maternal treatment in famliy	2	157587	78793	8.428	0.0006415	***
Residuals	55	514193	9349			
**Ribosomal protein A3**	Df	SS	MS	F value	Pr(>F)	
juvenile challenge	2	6.64E-06	3.32E-06	1.4962	0.233	
maternal treatment	1	8.34E-07	8.34E-07	0.3755	0.5426	
juvenile challenge * maternal treatment	2	3.27E-07	1.63E-07	0.0736	0.9292	
maternal treatment in famliy	2	2.00E-06	9.99E-07	0.4499	0.64	
Residuals	55	1.22E-04	2.22E-06			
**Serum amyloid A**	Df	SS	MS	F value	Pr(>F)	
juvenile challenge	2	0.0005022	0.00025112	2.0249	0.14173	
maternal treatment	1	0.0000798	0.00007978	0.6433	0.42595	
juvenile challenge * maternal treatment	2	0.0000617	0.00003086	0.2489	0.78057	
maternal treatment in famliy	2	0.0007723	0.00038613	3.1136	0.05236	.
Residuals	55	0.0068208	0.00012401			
**Tumor necrose factor beta**	Df	SS	MS	F value	Pr(>F)	
juvenile challenge	2	5.20E-08	2.60E-08	0.7422	0.480765	
maternal treatment	1	3.25E-07	3.25E-07	9.2896	**0.003538**	**
juvenile challenge * maternal treatment	2	2.34E-07	1.17E-07	3.3377	0.042846	*
maternal treatment in famliy	2	7.71E-08	3.86E-08	1.1017	0.339511	
Residuals	55	1.93E-06	3.50E-08			
