# Supplementary material for: Parental investment matters for maternal and offspring immune defense in the mouthbrooding cichlid Astatotilapia burtoni
Source: BMC Evol Biol. 2017 Dec 20;17:264. doi: 10.1186/s12862-017-1109-6 (PMC5738712; doi:10.1186/s12862-017-1109-6)
Supplement: Supplementary file 8 — Tukey HSD test on candidate genes from mouthbred juveniles: Posthoc test following significant results from two-way ANCOVA. P-values marked additionally in bold are in agreement with the results from the univariate analysis. (PDF 51 kb) [file 12862_2017_1109_MOESM8_ESM.pdf]

S8 Table: Tukey HSD test on candidate genes from mouthbred juveniles: Posthoc test following significant results from two-way ANCOVA. P-values marked in bold are in agreement with the results from the univariate analysis.

Interactive effects between maternal and juvenile treatment

Estrogen receptor B

	female naive : juvenile naive (fN : jN)				female Vibrio : juvenil naive (FV : jN)			
	diff	lower	upper	p value	diff	lower	upper	p value
female naive : juvenile naive (fN : jN)								
female Vibrio : juvenil naive (FV : jN)	18.13	-83.05	46.79	0.9619				
female naive : juvenile Tenacibacter (fN : jT)	33.49	-96.84	29.86	0.6283	15.36	-49.56	80.28	0.9815
female Vibrio : juvenile Tenacibacter (fV : jT)	28.16	-94.94	38.62	0.8136	10.03	-78.29	58.24	0.9980
female naive : juvenile Vibrio (fN : jV)	22.84	-82.70	37.03	0.8690	4.71	-56.81	66.22	0.9999
female Vibrio : juvenile Vibrio (fN : jN)	99.41	-168.45	-30.38	**0.0011**	81.28	-151.76	-10.81	**0.0149**

	female naive : juvenile Tenacibacter (fN : jT)				female Vibrio : juvenile Tenacibacter (fV : jT)			
	diff	lower	upper	p value	diff	lower	upper	p value
female naive : juvenile naive (fN : jN)								
female Vibrio : juvenil naive (FV : jN)								
female naive : juvenile Tenacibacter (fN : jT)								
female Vibrio : juvenile Tenacibacter (fV : jT)	5.33	-61.45	72.11	0.9999				
female naive : juvenile Vibrio (fN : jV)	10.65	-49.21	70.52	0.9950	5.32	-68.80	58.16	0.9999
female Vibrio : juvenile Vibrio (fN : jN)	65.92	-134.96	3.11	0.0693	71.26	-143.45	0.94	0.0550

	female naive : juvenile Vibrio (fN : jV)			
	diff	lower	upper	p value
female naive : juvenile naive (fN : jN)				
female Vibrio : juvenil naive (FV : jN)				
female naive : juvenile Tenacibacter (fN : jT)				
female Vibrio : juvenile Tenacibacter (fV : jT)				
female naive : juvenile Vibrio (fN : jV)				
female Vibrio : juvenile Vibrio (fN : jN)	76.58	-142.43	-10.73	**0.0137**

Complement component 1a

	female naive : juvenile naive (fN : jN)				female Vibrio : juvenil naive (FV : jN)			
	diff	lower	upper	p value	diff	lower	upper	p value
female naive : juvenile naive (fN : jN)								
female Vibrio : juvenil naive (FV : jN)	1.81E-04	-7.19E-04	3.56E-04	0.9180				
female naive : juvenile Tenacibacter (fN : jT)	4.79E-04	-1.00E-03	4.51E-05	0.0920	2.98E-04	-2.39E-04	8.36E-04	0.5787
female Vibrio : juvenile Tenacibacter (fV : jT)	1.23E-04	-6.76E-04	4.30E-04	0.9859	5.81E-05	-5.07E-04	6.23E-04	0.9996
female naive : juvenile Vibrio (fN : jV)	1.95E-04	-6.90E-04	3.01E-04	0.8539	1.37E-05	-4.96E-04	5.23E-04	1.0000
female Vibrio : juvenile Vibrio (fN : jN)	8.15E-04	-1.39E-03	-2.43E-04	**0.0013**	6.33E-04	-1.22E-03	-5.01E-05	**0.0258**

	female naive : juvenile Tenacibacter (fN : jT)				female Vibrio : juvenile Tenacibacter (fV : jT)			
	diff	lower	upper	p value	diff	lower	upper	p value
female naive : juvenile naive (fN : jN)								
female Vibrio : juvenil naive (FV : jN)								
female naive : juvenile Tenacibacter (fN : jT)								
female Vibrio : juvenile Tenacibacter (fV : jT)	3.56E-04	-1.97E-04	9.09E-04	0.4125				
female naive : juvenile Vibrio (fN : jV)	2.84E-04	-2.11E-04	7.80E-04	0.5422	7.18E-05	-4.54E-04	5.97E-04	0.9986
female Vibrio : juvenile Vibrio (fN : jN)	3.35E-04	-9.07E-04	2.36E-04	0.5182	6.92E-04	-1.29E-03	-9.39E-05	**0.0144**

	female naive : juvenile Vibrio (fN : jV)			
	diff	lower	upper	p value
female naive : juvenile naive (fN : jN)				
female Vibrio : juvenil naive (FV : jN)				
female naive : juvenile Tenacibacter (fN : jT)				
female Vibrio : juvenile Tenacibacter (fV : jT)				
female naive : juvenile Vibrio (fN : jV)				
female Vibrio : juvenile Vibrio (fN : jN)	6.20E-04	-1.16E-03	-7.46E-05	**0.0170**

Lysine specific demethylase

	female naive : juvenile naive (fN : jN)				female Vibrio : juvenil naive (FV : jN)			
	diff	lower	upper	p value	diff	lower	upper	p value
female naive : juvenile naive (fN : jN)								
female Vibrio : juvenil naive (FV : jN)	1.23E-04	-6.47E-04	4.01E-04	0.9820				
female naive : juvenile Tenacibacter (fN : jT)	3.14E-04	-8.25E-04	1.98E-04	0.4689	1.90E-04	-3.34E-04	7.15E-04	0.8906
female Vibrio : juvenile Tenacibacter (fV : jT)	2.39E-04	-7.78E-04	3.00E-04	0.7797	1.16E-04	-6.67E-04	4.35E-04	0.9891
female naive : juvenile Vibrio (fN : jV)	2.21E-04	-7.05E-04	2.62E-04	0.7557	9.82E-05	-3.99E-04	5.95E-04	0.9918
female Vibrio : juvenile Vibrio (fN : jN)	8.01E-04	-1.36E-03	-2.43E-04	**0.0011**	6.78E-04	-1.25E-03	-1.09E-04	**0.0108**

	female naive : juvenile Tenacibacter (fN : jT)				female Vibrio : juvenile Tenacibacter (fV : jT)			
	diff	lower	upper	p value	diff	lower	upper	p value
female naive : juvenile naive (fN : jN)								
female Vibrio : juvenil naive (FV : jN)								
female naive : juvenile Tenacibacter (fN : jT)								
female Vibrio : juvenile Tenacibacter (fV : jT)	7.46E-05	-4.65E-04	6.14E-04	0.9985				
female naive : juvenile Vibrio (fN : jV)	9.23E-05	-3.91E-04	5.76E-04	0.9930	1.77E-05	-5.30E-04	4.95E-04	1.0000
female Vibrio : juvenile Vibrio (fN : jN)	4.87E-04	-1.04E-03	7.02E-05	0.1197	5.62E-04	-1.14E-03	2.12E-05	**0.0651**

	female naive : juvenile Vibrio (fN : jV)			
	diff	lower	upper	p value
female naive : juvenile naive (fN : jN)				
female Vibrio : juvenil naive (FV : jN)				
female naive : juvenile Tenacibacter (fN : jT)				
female Vibrio : juvenile Tenacibacter (fV : jT)				
female naive : juvenile Vibrio (fN : jV)				
female Vibrio : juvenile Vibrio (fN : jN)	5.80E-04	-1.11E-03	-4.78E-05	**0.0250**

Effects of the single factor "juvenile treatment"

Calreticulin 1

	Control (PBS)				Tenebacter			
	diff	lower	upper	p value	diff	lower	upper	p value
Control (PBS)								
Tenacibacter	-23.60	-57.90	10.69	0.2310				
Vibrio	-34.83	-68.32	-1.34	**0.0398**	-11.23	-45.14	22.69	0.7065

Early growth response protein 1

	Control (PBS)				Tenebacter			
	diff	lower	upper	p value	diff	lower	upper	p value
Control (PBS)								
Tenacibacter	-29.84	-68.85	9.16	0.1655				
Vibrio	-54.01	-92.10	-15.93	**0.0034**	-24.17	-62.74	14.40	0.2948
